# Synergistic Effects of Co_3_O_4_-gC_3_N_4_-Coated ZnO Nanoparticles: A Novel Approach for Enhanced Photocatalytic Degradation of Ciprofloxacin and Hydrogen Evolution via Water Splitting

**DOI:** 10.3390/ma17051059

**Published:** 2024-02-25

**Authors:** Abniel Machín, Carmen Morant, Loraine Soto-Vázquez, Edgard Resto, José Ducongé, María Cotto, Pedro J. Berríos-Rolón, Cristian Martínez-Perales, Francisco Márquez

**Affiliations:** 1 Environmental Catalysis Research Lab, Division of Science, Technology and Environment, Cupey Campus, Universidad Ana G. Méndez, Cupey, PR 00926, USA; 2 Department of Applied Physics, Autonomous University of Madrid, and Instituto de Ciencia de Materiales Nicolás Cabrera, 28049 Madrid, Spain; c.morant@uam.es; 3 Materials Characterization Center Inc., Molecular Sciences Research Center, University of Puerto Rico, San Juan, PR 00926, USA; loraine.soto@mcc.com.pr (L.S.-V.); restoe@mcc.com.pr (E.R.); 4 Nanomaterials Research Group, Department of Natural Sciences and Technology, Division of Natural Sciences, Technology and Environment, Universidad Ana G. Méndez-Gurabo Campus, Gurabo, PR 00778, USA; jduconge@uagm.edu (J.D.); mcotto48@uagm.edu (M.C.); berriosp1@uagm.edu (P.J.B.-R.); cmartinez372@email.uagm.edu (C.M.-P.)

**Keywords:** photodegradation, photocatalytic hydrogen evolution, ciprofloxacin

## Abstract

This research evaluates the efficacy of catalysts based on Co_3_O_4_-gC_3_N_4_@ZnONPs in the degradation of ciprofloxacin (CFX) and the photocatalytic production of H_2_ through water splitting. The results show that CFX experiences prompt photodegradation, with rates reaching up to 99% within 60 min. Notably, the 5% (Co_3_O_4_-gC_3_N_4_)@ZnONPs emerged as the most potent catalyst. The recyclability studies of the catalyst revealed a minimal activity loss, approximately 6%, after 15 usage cycles. Using gas chromatography–mass spectrometry (GC-MS) techniques, the by-products of CFX photodegradation were identified, which enabled the determination of the potential degradation pathway and its resultant products. Comprehensive assessments involving photoluminescence, bandgap evaluations, and the study of scavenger reactions revealed a degradation mechanism driven primarily by superoxide radicals. Moreover, the catalysts demonstrated robust performance in H_2_ photocatalytic production, with some achieving outputs as high as 1407 µmol/hg in the visible spectrum (around 500 nm). Such findings underline the potential of these materials in environmental endeavors, targeting both water purification from organic pollutants and energy applications.

## 1. Introduction

The twin challenges of unsustainable energy consumption and the alarming rise in antibiotic-resistant bacteria are converging to create a complex, multifaceted crisis that threatens the future of both environmental stability and public health. Predominantly reliant on fossil fuel sources such as coal, oil, and natural gas, the current global energy landscape contributes significantly to climate change and environmental degradation [1,2]. This reliance manifests in increased greenhouse gas emissions, accelerated global warming, and the subsequent threats to ecosystems and biodiversity [2]. Environmental instability further exacerbates the existing public health issues, ranging from respiratory diseases caused by air pollution to more frequent and severe natural disasters that put communities at risk [3]. As a clean and high-energy-density fuel, hydrogen offers a compelling pathway for transitioning away from fossil fuel dependency, especially given its capability for seamless integration into existing energy infrastructures for both the electricity and transportation sectors [4].

On the other hand, the growing prevalence of antibiotic-resistant bacteria is an escalating public health emergency that undermines decades of medical advancements in treating bacterial infections [5]. The improper disposal of antibiotics, especially into water systems, not only contaminates natural water sources but also facilitates the development of drug-resistant strains of bacteria [6]. These ‘superbugs’ are progressively rendering traditional antibiotic treatments ineffective, leading to longer hospital stays, higher medical costs, and increased mortality rates [6]. Thus, the issues of unsustainable energy production and antibiotic resistance are not isolated; they intersect and magnify the existing challenges in maintaining environmental balance and ensuring public health safety. Innovative and holistic solutions are urgently required to address these pressing global concerns.

One way to produce hydrogen and degrade antibiotics from the water is the use of photocatalysts. Photocatalysis is a process that leverages the excitation of a semiconductor material by photons to facilitate redox reactions [7]. Upon irradiation with light energy equal to or greater than its bandgap, the semiconductor generates electron–hole pairs [7]. In an aqueous medium, these photogenerated electrons and holes migrate to the surface of the catalyst, where they participate in redox reactions. Electrons reduce adsorbed species, like oxygen molecules or protons, while holes oxidize adsorbates like organic pollutants or water molecules [8]. The efficiency of this process is significantly influenced by factors such as the bandgap energy, surface area, charge carrier lifetime, and the rate of recombination, which can be fine-tuned through material engineering, doping, or through the use of co-catalysts [9,10].

Considering these challenges, substantial progress has been made to create more efficient and effective photocatalysts. One of the most promising developments in this area involves the use of heterostructured composites made up of materials such as zinc oxide nanoparticles (ZnO), cobalt oxide (CoO), and graphitic carbon nitride (g-C_3_N_4_) [11,12]. The combination of the unique advantages of each material could offer a path to both the degradation of antibiotics in water and the photocatalytic splitting of water for hydrogen production.

For example, Long et al. [13] developed a heterojunction and oxygen vacancy modification of a ZnO nanorod array photoanode for photoelectrochemical water splitting. The authors explained that CoOx nanoparticles served the dual function of forming a p-n heterojunction to facilitate the separation of photogenerated carriers and acted as a co-catalyst to decrease the water oxidation barrier. Furthermore, the group argues that the oxygen vacancies increased the number of active redox sites and acted as hole traps, enabling their migration to the electrode/electrolyte interface. Another research group [14] reported the use of ZnO/CoO for the degradation of methylene blue. The group reported that the incorporation of CoO modified the bandgap of the photocatalysts allowing them to degrade methylene blue under visible light in under 3 h.

The use g-C_3_N_4_@ZnO for photocatalytic hydrogen production has been reported by Zada et al. [15]. The group fabricated ZnO with 2D g-C_3_N_4_ nanosheets and the obtained nanocomposites were applied for photocatalytic hydrogen generation from water under visible light illumination (λ > 420 nm). The results showed that the optimized g-C_3_N_4_@ZnO nanocomposite produced 70 μmol hydrogen gas in 1 h compared to 8 μmol by pure g-C_3_N_4_ under identical illumination conditions in the presence of methanol. The authors attribute the enhancement in the production to a more efficient charge separation. In the case of organic compounds, Thi et al. [16] constructed a g-C_3_N_4_@ZnO composite and measured its photocatalytic activity by the degradation of the antibiotic ciprofloxacin. The authors reported an 84.36% degradation after one hour and a high degradation efficiency after three recyclability tests. The high photocatalytic degradation is attributed to the efficient separation of photogenerated electron–hole pairs.

As of the present state of scientific research, the deployment of Co_3_O_4_-gC_3_N_4_@ZnONPs composites for the dual purpose of hydrogen production through water splitting and the photodegradation of antibiotics, with a specific focus on ciprofloxacin, remains unexplored. This gap in the literature signals a significant opportunity for advancing our understanding of photocatalytic materials and their multifunctional applications. Accordingly, this study is designed to pioneer the development, synthesis, and in-depth characterization of novel heterostructured Co_3_O_4_-gC_3_N_4_@ZnONPs composites. Our objectives extend beyond mere synthesis; we aim to rigorously assess these composites’ photocatalytic activities in the context of hydrogen generation via water splitting and the efficient degradation of ciprofloxacin, a widely used antibiotic.

The rationale behind selecting ciprofloxacin as a target for degradation stems from its prevalent usage and resultant environmental persistence, which poses emerging challenges to aquatic ecosystems and potentially human health. Concurrently, the quest for sustainable hydrogen production methods has intensified, spotlighting water splitting as a promising avenue for renewable energy. By addressing these two critical areas, our research endeavors to contribute significantly to environmental preservation and the development of green energy solutions.

## 2. Materials and Methods

### 2.1. Materials

All the reactants were used as received and the solutions were prepared using deionized water (18.2 MΩcm^−1^ at 25 °C, Barnstead GenPure water purificationsystems from ThermoScientific, (Waltham, MA, USA)). The synthesis of the ZnONPs required the use of zinc acetate (Zn(C_2_H_3_O_2_)_2_.2H_2_O; 98.99%) and sodium hydroxide (NaOH; 99.0%), both acquired by Sigma Aldrich (Milwaukee, WI, USA). The synthesis of gC_3_N_4_ and Co_3_O_4_-gC_3_N_4_ required urea (NH_2_CONH_2_; ReagentPlus; ≥99.5%) and cobalt(II) acetate tetrahydrate (Co(C_2_H_3_O_2_)_2_.4H_2_O, ACS reagent; >98.0%), provided by Sigma Aldrich (Milwaukee, WI, USA). Cobalt(II,III) oxide (Co_3_O_4_, ≥99.99%), ciprofloxacin (C_17_H_18_FN_3_O_3_; 98%), hydrogen peroxide (H_2_O_2_; 35% *w*/*w*), ethylenediaminetetraacetic acid (EDTA-Na_2_; ACS reagent; 99.4–100.6% powder), methanol (CH_3_OH; HPLC grade; >99.9%), and 0.45 μm syringe filters were provided by Sigma Aldrich (Milwaukee, WI, USA).

### 2.2. Synthesis of ZnONPs

The production method for zinc oxide nanoparticles (ZnONPs) can be found in prior work [7]. The procedure involves blending 25 mL of a 0.2 M aqueous solution of zinc acetate dihydrate (Zn(C_2_H_3_O_2_)_2_.2H_2_O) with 50 mL of deionized water and heating the mixture to 60 °C. Once the target temperature was achieved, 25 mL of a 4 M sodium hydroxide (NaOH) solution was added. The mixture was maintained at 60 °C and stirred continuously for 2 h. Following this, the mixture was allowed to cool to room temperature. The resulting precipitate was then isolated via centrifugation and subjected to multiple washes until the rinse water attained a neutral pH level. The product was finally gathered and air-dried for an extended period at 60 °C.

### 2.3. Synthesis of gC_3_N_4_

For the synthesis of graphitic carbon nitride (g-C_3_N_4_), 20 g of analytical-grade urea was transferred to an alumina crucible [17]. The crucible was inserted into a tube furnace preheated to 200 °C under a nitrogen atmosphere to ensure a contaminant-free environment. The temperature was ramped to 550 °C at a rate of 5 °C/min and held constant for 3 h, with nitrogen gas flowing at 50 mL/min to maintain an inert atmosphere. After the dwell time, the sample was allowed to cool to room temperature under a nitrogen flow and subsequently ground for further use.

### 2.4. Synthesis of Co_3_O_4_-gC_3_N_4_

The Co_3_O_4_-gC_3_N_4_ composite was synthesized through a straightforward, single-step calcination process involving a mixture of urea and cobalt acetate dihydrate, according to the procedure adapted from Suhag et al. [18]. Specifically, 20 g of urea and 2 g of cobalt acetate were homogeneously dispersed in 5 mL of water and placed in an alumina crucible. The crucible was then partially sealed and positioned in a tube furnace. The temperature was ramped up to 550 °C at a heating rate of 5 °C/min while maintaining an airflow rate of 50 mL/min, and was held at this calcination temperature for 3 h. Subsequently, the sample was allowed to cool under a nitrogen flow of 50 mL/min for 2 h. Upon completion, the synthesized material was manually ground in an agate mortar, resulting in a dark black powder. The chemical composition of the synthesized material was analyzed using XPS, revealing the following atomic percentages: cobalt 12%, oxygen 22%, and carbon 66%.

### 2.5. Preparation of Adducts with ZnONPs

In the current study, two types of adducts were prepared: gC_3_N_4_@ZnONPs and Co_3_O_4_-gC_3_N_4_@ZnONPs. For each type, appropriate amounts of the respective components were precisely weighed and then dispersed in deionized water. To ensure maximum homogenization of the components within the adducts, the aqueous suspensions were subjected to ultrasonic treatment for 10 min. Following ultrasonication, the suspensions were centrifuged at 3000 rpm for 5 min. The precipitates were then collected with ethanol, and the samples were left to dry in an oven at 60 °C overnight.

### 2.6. Characterization Techniques

The surface area of the catalysts was evaluated through Brunauer–Emmett–Teller (BET) analysis utilizing a Micrometrics ASAP 2020 instrument, employing nitrogen gas adsorption isotherms at a temperature of 77 K. Morphological attributes of the composite materials were assessed via field emission scanning electron microscopy (FESEM) on an FEI Verios 460 L instrument. High-resolution transmission electron microscopy (HRTEM) examinations were conducted using a JEOL JEM 3000F microscope, operating at 300 kV. The crystalline structures of the catalysts were investigated through X-ray diffraction (XRD) on a Bruker D8 Advance instrument, operating at 40 kV and 40 mA. Raman spectral analysis was executed on a DXR Thermo Raman microscope, utilizing a 532 nm laser source at a power setting of 5 mW and a resolution of 5 cm^−1^. X-ray photoelectron spectroscopy (XPS) data were acquired with an ESCALAB 220i-XL spectrometer, using non-monochromatic Mg Kα radiation of a twin anode at 20 mA and 12 kV. Bandgap energies of the catalysts, obtained from the plot of the Kubelka–Munk function versus the energy of the absorbed light [19], and antibiotic degradation rates were ascertained using a Perkin Elmer Lambda 365 UV–vis spectrophotometer. Photoluminescence measurements were performed on an Edinburgh FS900 fluorescence spectrometer. Intermediates from the photodegradation process were examined using gas chromatography/mass spectrometry (GC-MS) on a GC 2010 Plus-QP2020 GC-MS instrument (Shimadzu Corporation, Japan). Compounds were fractionated using a 30 m × 0.25 mm i.d. capillary column (Rtx-5MS, Restek Corporation, Bellefonte PA, USA) with helium (99.999% purity) serving as the carrier gas.

### 2.7. Photocatalytic Degradation Experiments

The photodegradation assays involved the formulation of a 10 μM CFX solution, which was mixed with 1.1 g/L of the selected catalyst. The pH value of the resultant mixture was then adjusted to 7 utilizing either sodium hydroxide (NaOH) or hydrochloric acid (HCl). The solution was kept in the dark for 30 min under constant stirring to attain adsorption/desorption equilibrium with the catalyst. Following this, a 1 mL aliquot of a 0.01% hydrogen peroxide (H_2_O_2_) solution was introduced, and the mixture was aerated continuously to ensure oxygen availability. The solution was then exposed to a solar simulator, equipped with dual white light bulbs (100 watts and ca. 6300 lx). Upon activation of the irradiation system, the reaction proceeded for 60 min at 22 °C, with 5 mL samples being extracted at 10 min intervals. These samples were subsequently passed through 0.45 μm membrane filters to eliminate the catalyst and were analyzed using UV–visible spectroscopy.

For the detailed examination of by-products produced during the photodegradation process, aliquots were sampled at varied intervals throughout the reaction duration. Prior to analysis, these samples were filtered to remove any residual catalyst. Each aliquot was subsequently diluted in 50 mL of deionized water, and the organic compounds were then extracted with ethyl acetate using a liquid–liquid extraction method. Once extracted, the organic phase was concentrated to dryness with the aid of a rotary evaporator. The resulting residue was reconstituted in 5 mL of methanol for subsequent analysis. Quantitative and qualitative assessments were performed by GC-MS, where a 1 µL aliquot of the prepared sample was introduced for chromatographic separation and mass spectral detection. For comprehensive analysis of the contaminant, a series of four injections were executed, comprising three test samples and one blank, with helium employed as the carrier gas to facilitate the chromatographic process.

### 2.8. Photocatalytic Hydrogen Production Experiments

The experimental setup designed for investigating hydrogen generation through the process of water splitting involved the mixture of 50 mg of the selected catalyst with 100 mL of deionized water within a 250 mL quartz-based reaction chamber. Subsequently, sacrificial electron donor solutions, specifically sodium sulfite (Na_2_SO_3_) at a concentration of 0.02 M and sodium sulfide (Na_2_S) at 0.4 M, were introduced into the reaction mixture. The entire reaction assembly was maintained at 20 °C and subjected to a nitrogen (N_2_) purge for 30 min to eliminate any residual oxygen and other gaseous impurities. After the purging phase, the reaction mixture was subjected to UV–vis light irradiation with an intensity of 120 mW.cm^−2^ (in the absence of filters). Irradiation was performed at predetermined wavelengths of 220 nm, 320 nm, 400 nm, 500 nm, 600 nm, and 700 nm using specific cut-off filters. The irradiation was sustained for a period of 2 h to facilitate the catalytic splitting of water molecules and the consequent generation of hydrogen. The evolved hydrogen was subsequently captured, and its volume was quantitatively assessed utilizing a gas chromatographic system equipped with a thermal conductivity detector (GC–TCD). Specifically, a Perkin-Elmer Clarus 600 instrument was employed for this purpose.

## 3. Results and Discussion

### 3.1. Characterization of the Catalysts

**Figure 1:**
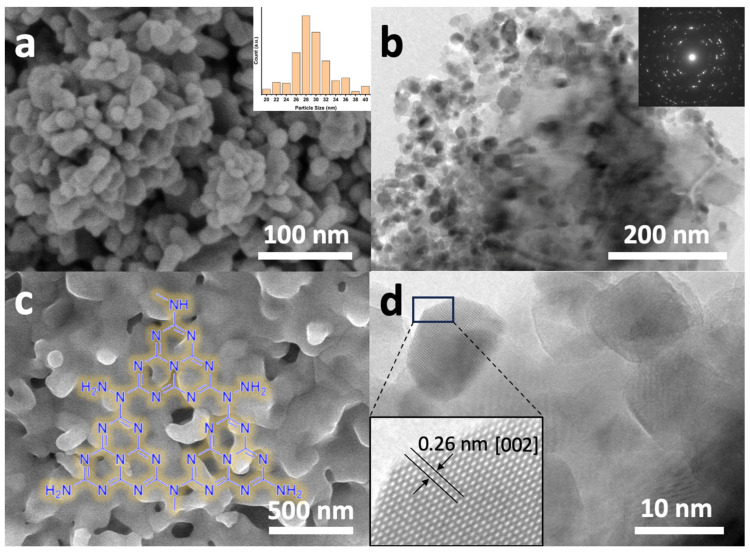
Electron microscopy characterizations of various catalyst components: (**a**) Field emission scanning electron microscopy (FESEM) image of ZnONPs, with the inset showing the average particle size distribution.; (**b**) HRTEM image of ZnONPs with an inset showing the corresponding SAED pattern; (**c**) FESEM image of Co_3_O_4_-gC_3_N_4_ composite; (**d**) HRTEM image of ZnONPs, revealing an enlarged detail of a single nanoparticle exhibiting a lattice fringe of 0.26 nm, characteristic of the ZnO wurtzite structure.

The morphological characteristics of the synthesized ZnONPs were investigated using scanning electron microscopy, as depicted in Figure 1. The ZnO nanoparticles (ZnONPs), shown in Figure 1a, display a relatively uniform size distribution with diameters ranging approximately from 26 to 32 nm. To determine the average particle size, the ImageJ software (version 1.53m, https://imagej.nih.gov/ij) was utilized, analyzing various areas of the SEM images, which yielded an average value of 28 nm (see inset of Figure 1a). Figure 1b presents a high-resolution transmission electron microscopy (HR-TEM) image of the same material, revealing its high degree of homogeneity and crystalline nature, as evidenced by the corresponding selected area electron diffraction (SAED) pattern shown in the inset.

Figure 1c presents the scanning electron microscopy (SEM) image of the Co_3_O_4_-gC_3_N_4_ composite obtained through the thermal treatment of a mixture of urea and cobalt acetate dihydrate. As can be observed, the sample exhibits an irregular morphology, characterized by small protrusions and an apparently low-porosity structure formed by Co_3_O_4_, which is likely embedded within the laminar framework of gC_3_N_4_. Given that the delamination of gC_3_N_4_ necessitates acidic treatment under harsh conditions, which would adversely impact Co_3_O_4_, the material was employed in its as-synthesized state, subsequent to grinding in an agate mortar. The HRTEM image of the ZnONPs is also shown in Figure 1d. The material is observed to be highly crystalline. The inset features a magnified region of the image, revealing the atomic structure of the material with a lattice spacing of 0.26 nm, which has been attributed to ZnO with a wurtzite structure [20].

**Figure 2:**
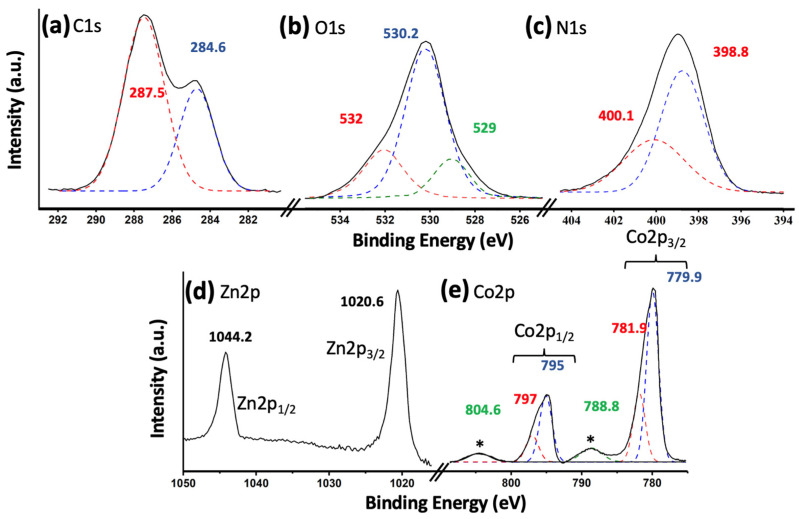
XPS core level spectra for 5% (Co_3_O_4_-gC_3_N_4_)@ZnONPs: C1s (**a**); O1s (**b**); N1s (**c**); Zn2p (**d**); and Co2p (**e**). The asterisks in (**e**) represent the characteristic satellite peaks of the Co_3_O_4_ species.

Figure 2 shows the X-ray photoelectron spectroscopy (XPS) results for the most active catalyst, namely 5% (Co_3_O_4_-gC_3_N_4_)@ZnONPs. Figure 2a shows the C1s transition, characterized by having two peaks at 284.6 eV and 287.5 eV, respectively. The peak at 284.6 eV is commonly attributed to sp^2^ hybridized carbon–carbon (C-C) bonds, while the peak at 287.5 eV is associated with sp-hybridized carbon in nitrogen-containing aromatic rings (N-C=N) [21,22]. These peaks signify the predominant carbon species present in graphitic carbon nitride (gC_3_N_4_). Figure 2b depicts the O1s transition, featuring a prominent peak approximately centered at 530.2 eV. This peak is attributed to O^2−^ species within the ZnO lattice [23,24]. Additionally, two subsidiary peaks are observed at approximately 529.0 eV and 532.1 eV. These are ascribed to lattice oxygen in Co_3_O_4_ and O^2−^ species in oxygen-deficient zones of the ZnO structure, respectively [11,25]. In Figure 2c, the binding energies for the N1s spectrum are identified as 398.8 eV and 400.1 eV. The principal peak, centered at 398.8 eV, arises from sp^2^-hybridized nitrogen atoms participating in triazine rings, which are the dominant nitrogen-containing structures in graphitic carbon nitride (gC_3_N_4_) [26]. Conversely, the minor peak located at 400.1 eV is attributed to tertiary nitrogen groups, denoted as N-(C)_3_ [26]. Figure 2d displays the Zn2p transition, featuring two distinct peaks at 1020.6 eV and 1044.2 eV. These peaks are associated with the Zn2p_3/2_ and Zn2p_1/2_ electronic states, respectively [27]. Additionally, they exhibit a characteristic spin-orbit coupling of 23.6 eV, which is indicative of the Zn^2+^ oxidation state [28]. In Figure 2e, the Co2p transition is characterized by two primary peaks centered around 780 eV and 795 eV, corresponding to the Co2p_3/2_ and Co2p_1/2_ transitions, respectively [27]. These peaks were subsequently deconvoluted. The 15 eV energy separation between the Co2p_1/2_ and Co2p_3/2_ peaks indicates the simultaneous presence of the Co^2+^ and Co^3+^ oxidation states, suggestive of a Co_3_O_4_ composition [29]. The peaks at 779.9 eV and 795 eV were ascribed to Co^3+^, while those observed at 781.9 eV and 797 eV were attributed to Co^2+^ [29]. Additionally, satellite peaks were observed at 788.8 eV and 804.6 eV, which are characteristic features of Co_3_O_4_ [30].

**Figure 3:**
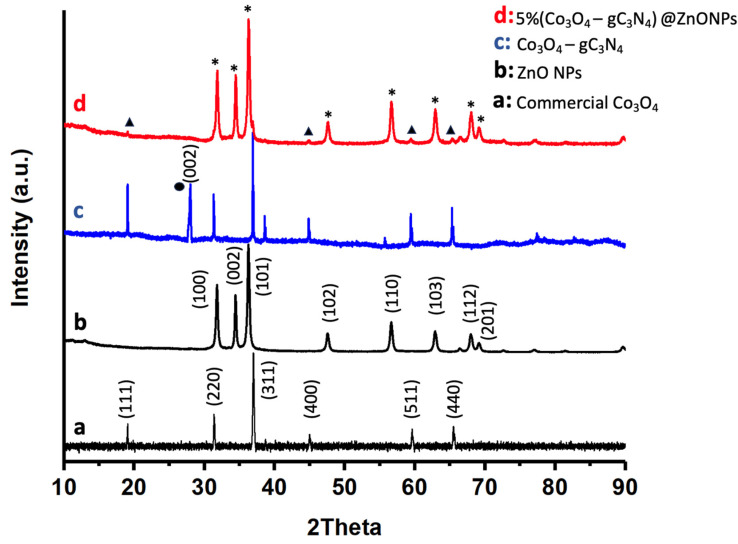
XRD patterns of commercial Co_3_O_4_ (**a**); ZnONPs (**b**); Co_3_O_4_-gC_3_N_4_ (**c**); and 5% (Co_3_O_4_-gC_3_N_4_)@ZnONPs (**d**). The black circle represents the reflections of gC_4_N_3_, while the asterisks and triangles represent signals from ZnO or Co_3_O_4_, respectively.

The crystalline nature of the most active catalyst, as well as its various components, was investigated using X-ray diffraction (XRD) as shown in Figure 3. For comparative purposes, Figure 3 also displays the diffraction pattern of commercial Co_3_O_4_ (Figure 3a). As can be observed, this compound exhibits characteristic peaks that have been attributed to Co_3_O_4_ with a pure spinel structure (JCPDS card No. 00-042-1467) [31]. The diffraction peaks for ZnONPs, as depicted in Figure 3b, can be definitively assigned to the hexagonal wurtzite phase of ZnO (JCPDS card No. 043-0002) [32]. These peaks are the predominant features in the 5% (Co_3_O_4_-gC_3_N_4_)@ZnONPs catalyst, as evidenced in Figure 3d. The adduct (Co_3_O_4_-gC_4_N_3_), as depicted in Figure 3c, is characterized by XRD peaks that align with the reference material shown in Figure 3a. Additionally, an extra peak around 27°, marked with a black circle, has been attributed to the (002) reflection of gC_4_N_3_ [33]. Figure 3d presents the diffractogram of the 5% (Co_3_O_4_-gC_4_N_3_)@ZnONPs catalyst, where signals originating from ZnO or Co_3_O_4_ are indicated by asterisks and triangles, respectively.

**Figure 4:**
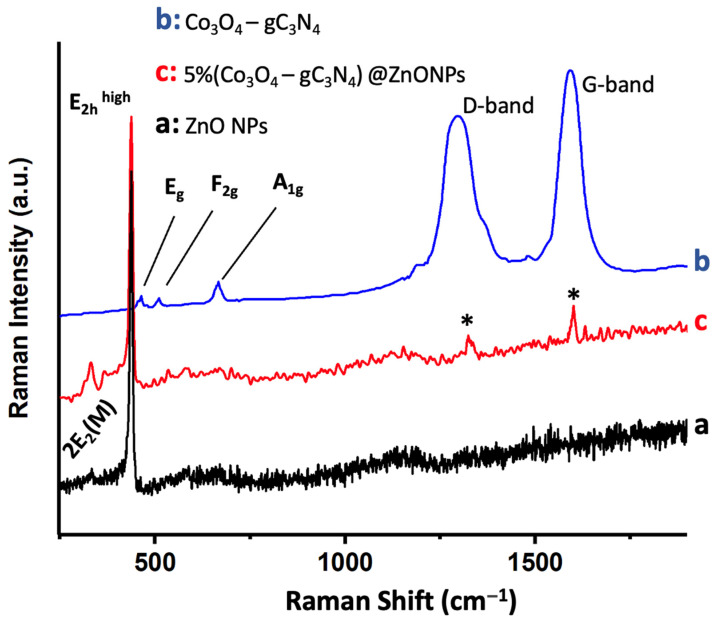
Raman spectra of ZnONPs (**a**); Co_3_O_4_-gC_3_N_4_ (**b**); and 5% (Co_3_O_4_-gC_3_N_4_)@ZnONPs (**c**). Asterisks in (**c**) correspond to gC3N4.

Figure 4 shows the Raman spectrum of 5% (Co_3_O_4_-gC_3_N_4_)@ZnONPs, along with the spectra of Co_3_O_4_-gC_3_N_4_ and ZnONPs. The ZnONPs spectrum (Figure 4a) displays a peak around 437 cm^−1^, attributed to the E_2h_^high^ mode, and a fainter peak near 333 cm^−1^, assigned to the 2E_2_(M) mode [34,35]. The Co_3_O_4_-gC_3_N_4_ adduct exhibits several subdued peaks around 482 cm^−1^, 522 cm^−1^, and 690 cm^−1^, which are associated with the E_g_, F_2g_, and A_1g_ vibrational modes of Co_3_O_4_, respectively [36,37]. The A_1g_ mode corresponds to the octahedral CoO_6_ symmetry, while the other two modes (E_g_ and F_2g_) are linked to tetrahedral CoO_4_ sites [36,37]. The most pronounced Raman peaks observed in the adduct around 1297 cm^−1^ and 1592 cm^−1^ are attributed to the D and G bands of gC_3_N_4_, respectively (see Figure 4b) [10]. The D band is related to the potential presence of sp^3^ carbon, arising from structural imperfections and dislocations, whereas the G band signifies the presence of sp^2^ carbon [10]. Lastly, the Raman spectrum of the most active catalyst, 5% (Co_3_O_4_-gC_3_N_4_)@ZnONPs (see Figure 4c), reveals the characteristic peaks of the predominant component (i.e., ZnONPs) and much fainter peaks (marked with asterisks) corresponding to gC_3_N_4_.

To rationalize the photocatalytic activity of the various materials, BET surface areas were determined (Table S1). As observed, the catalytic support (ZnONPs) exhibits a relatively high surface area for such a material type, registering at 76 m^2^/g. The adduct (Co_3_O_4_-gC_3_N_4_) displayed a significantly higher specific surface area of 187 m^2^/g, attributable to the layered nature of gC_3_N_4_. Upon incorporating varying amounts of the adduct to the ZnONPs support, catalysts with specific surface areas ranging from 93 m^2^/g for 1% (Co_3_O_4_-gC_3_N_4_)@ZnONPs to 231 m^2^/g for 10% (Co_3_O_4_-gC_3_N_4_)@ZnONPs were obtained. At first glance, this might suggest a potential increase in catalytic activity for materials with a higher proportion of the adduct in their composition. However, as will be discussed in subsequent sections analyzing the obtained results, the most active catalyst is not necessarily the one with the highest proportion of Co_3_O_4_-gC_3_N_4_.

**Figure 5:**
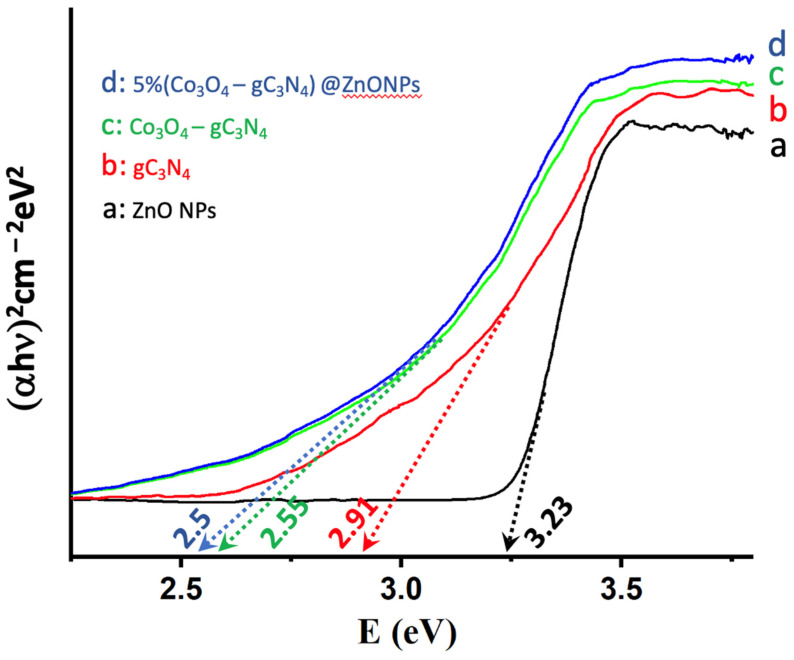
Tauc plots of (αhn)^2^ versus energy (eV), and determination of the bandgap energy of ZnONPs (**a**); gC_3_N_4_ (**b**); Co_3_O_4_-gC_3_N_4_ (**c**); and 5% (Co_3_O_4_-gC_3_N_4_)@ZnONPs (**d**).

The ability of catalysts to absorb radiation is pivotal for their catalytic performance, prompting an analysis of the various systems using Tauc plots [19]. As illustrated in Figure 5, the absorption characteristics of ZnONPs, gC_3_N_4_, Co_3_O_4_-gC_3_N_4_, and the most active catalyst, 5% (Co_3_O_4_-gC_3_N_4_)@ZnONPs, were investigated. It is noteworthy that ZnONPs primarily absorb in the UV domain, displaying a bandgap of 3.23 eV. Conversely, gC_3_N_4_ exhibits an absorption edge at approximately 2.91 eV, already within the visible spectrum. In contrast, the bandgap for the composite (Co_3_O_4_-gC_3_N_4_) underwent a significant shift to 2.55 eV, translating to an absorption onset in the visible region (ca. 486 nm). The catalyst, depicted in Figure 5c, showcases an even more pronounced red-shifted bandgap in the visible spectrum at 2.5 eV. As will be elaborated upon later, these findings elucidate the behavior of the catalysts. Specifically, in hydrogen production experiments under varied wavelength irradiation, they underscore the enhanced activity of the catalysts in the visible range.

### 3.2. Photocatalytic Degradation of CFX

Prior to the CFX photodegradation experiments, a series of preliminary tests were conducted to determine the ideal reaction conditions. An initial assessment of the optimal pH was undertaken (Figure S1), employing the most effective catalyst, 5% (Co_3_O_4_-gC_3_N_4_)@ZnONPs. Photodegradation measurements of CFX were performed across a pH range from 5 to 10. The results indicated that the highest photodegradation rate was achieved at pH = 7, establishing this as the optimal pH for the process. The CFX concentration was also optimized, spanning a range from 2 µM to 50 µM, using two distinct catalysts (see Figure S2). As observed, despite the differing activities of the two catalysts, the peak photodegradation rate was noted at an initial CFX concentration of 10 µM. This concentration was thus set as the standard for all subsequent experiments. The catalyst loading in the reaction mixture was further evaluated (Figure S3). Two different catalysts were used, with catalyst loadings varying between 0.5 g/L and 1.5 g/L. The findings revealed that the highest photodegradation rate for both catalysts was achieved at a catalyst loading of 1.1 g/L. In light of the previously presented results, the conditions were optimized to a pH of 7, an initial CFX concentration of 10 mM, and a catalyst loading of 1.1 g/L.

**Figure 6:**
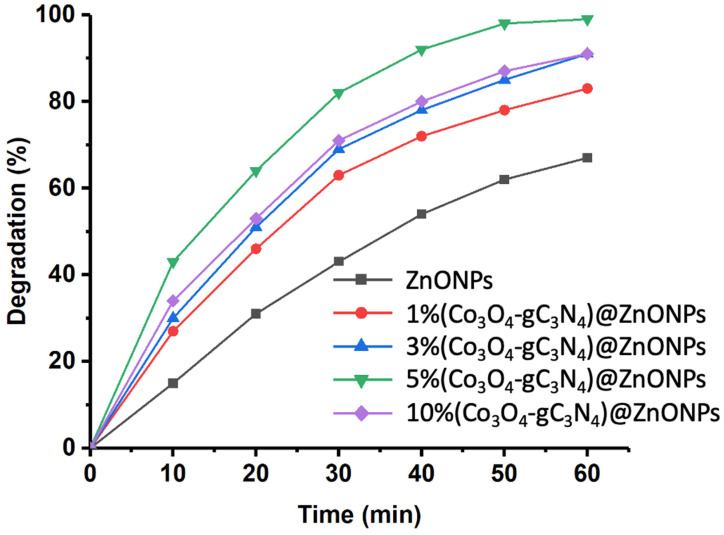
Rate of CFX photodegradation over time with various assessed catalysts.

Figure 6 shows the photodegradation results of CFX using the different catalysts studied. The investigation was conducted over a total reaction time of 60 min. As can be observed, ZnONPs achieves over 60% degradation of the initial CFX after 60 min. This performance is significantly enhanced in the presence of increasing amounts of Co_3_O_4_-gC_3_N_4_. For instance, after 60 min of reaction, the photodegradation achieved with the 1% (Co_3_O_4_-gC_3_N_4_)@ZnONPs catalyst increases to levels exceeding 80%. This photodegradation rate further elevates with the subsequent catalysts. However, similar behaviors were observed between the catalysts with 3% (Co_3_O_4_-gC_3_N_4_) and 10% (Co_3_O_4_-gC_3_N_4_), reaching degradation levels close to 90%. The most active catalyst was 5% (Co_3_O_4_-gC_3_N_4_)@ZnONPs, yielding a degradation rate close to 98% within 60 min. The results suggest a potential saturation effect, which influences the performance of the catalyst with the highest percentage of Co_3_O_4_-gC_3_N_4_, reducing its efficiency.

To explore the kinetic patterns of CFX photodegradation, we assessed the pseudo-first-order kinetics by plotting −ln (C/C_0_) against irradiation time (see Figure S4). The outcomes are detailed in Table S2, which demonstrates that the apparent rate constant of the catalysts rises with an increased proportion of Co_3_O_4_-gC_3_N_4_ in the ZnONPs. However, the catalyst with the highest Co_3_O_4_-gC_3_N_4_ loading, specifically 10% (Co_3_O_4_-gC_3_N_4_)@ZnONPs, exhibits a distinct pattern, as discussed in the context of the findings presented in Figure 6. This trend might also be correlated, at least partially, with the BET surface areas of these catalysts. Hence, as displayed in Table S1, materials with elevated BET surface areas tend to exhibit superior catalytic performance, characterized by increased apparent rate constants.

To delve deeper into the photocatalytic performance of the most active catalyst, namely 5% (Co_3_O_4_-gC_3_N_4_)@ZnONPs, both control experiments and recyclability assessments were conducted. The control data for CFX is presented in Figure S5. Upon a reaction time of 60 min, CFX appeared highly persistent, as underscored by the negligible changes in its concentration over time without the presence of either a catalyst or a light source. In an oxygen-depleted environment, enhanced CFX degradation is evident, though the degradation extent after 60 min remains inconsequential. From the control data, it is evident that photocatalysis is the predominant degradation mechanism, highlighting that sole catalysis or mere photolysis is entirely ineffective in breaking down CFX. The recyclability results for 5% (Co_3_O_4_-gC_3_N_4_)@ZnONPs over 15 cycles are shown in Figure S6. Post each cycle, the catalyst was retrieved using centrifugation (3000 rpm for 15 min). Following the removal of the supernatant, the catalyst was subjected to three washes with deionized water and a single wash with ethanol (employing centrifugation–sonication sequences), and then dried for a minimum of 3 h at 60 °C. The conditions for CFX degradation were maintained at previously defined optimal values. As evident in Figure S6, the degradation exhibited a mild decrease after each cycle, transitioning from 99% post the first cycle to 93% after the fifteenth cycle. To discern the reason for the efficiency decline, XPS-based elemental analyses of the catalyst were conducted before use and after the 15th cycle (not shown). The initial atomic composition was as follows: cobalt 3%, carbon 15%, zinc 31%, and oxygen 51%. Following the 15th cycle, the composition shifted to cobalt 2%, carbon 17%, zinc 28%, and oxygen 53%. The Co/Zn ratio decreased from 0.097 to 0.071, suggesting cobalt leaching, potentially explaining the ~6% activity drop.

**Figure 7:**
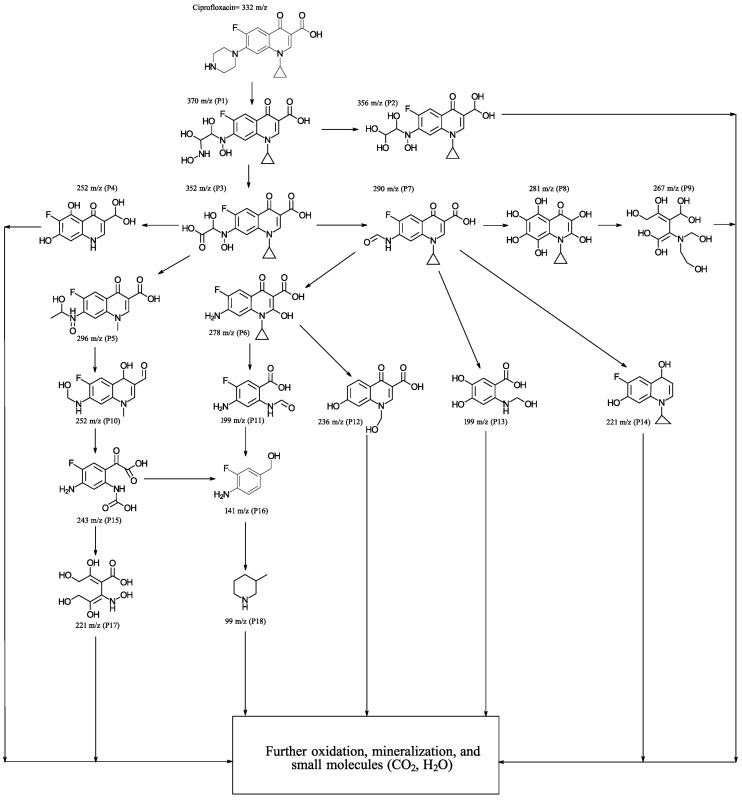
Proposed degradation pathways for the photocatalytic degradation of CFX with 5% (Co_3_O_4_-gC_3_N_4_)@ZnONPs.

To delve into the degradation process, monitoring was conducted using GC-MS. This involved identifying the reaction intermediates after subjecting the reaction mixture to the same irradiation conditions previously described, specifically after 20 min of irradiation. Following irradiation, an aliquot was taken, subsequently filtered through a membrane filter to remove the catalyst, and processed as outlined in the experimental section. The various peaks detected by chromatography were identified, and the results are displayed in Figure 7. As can be observed, the degradation pathway involves an initial stage where the precursor P1 is generated by cleavage of the pyrazine ring, through which the precursor P2 is formed, due to the loss of an amino group. From P1 and P2, various oxidation processes occur and residues are eliminated, although the quinoline and cyclopropyl rings initially continue to be maintained. In the later stages of the degradation process, intermediates are detected that are consistent with the cleavage of the quinoline ring and subsequent hydroxylation (P9), which can rapidly undergo further oxidation and mineralization processes, resulting in simpler molecules. From P3, various intermediates are generated, one of which (P15) has been previously detected [38]. The intermediate P7, reported previously in the literature [39], as observed in the potential degradation pathway in Figure 7, generates various oxidation products that, in general, retain an aromatic ring derived from the quinoline nucleus. From the intermediate P11, compounds P16 and P18 are obtained, which have already been reported in the literature [5,40]. The smaller intermediates are much less recalcitrant to degradation; therefore, they undergo very rapid transformation processes, resulting in much smaller molecules that are more challenging to identify.

### 3.3. Proposed Photocatalytic Degradation Mechanism

**Figure 8:**
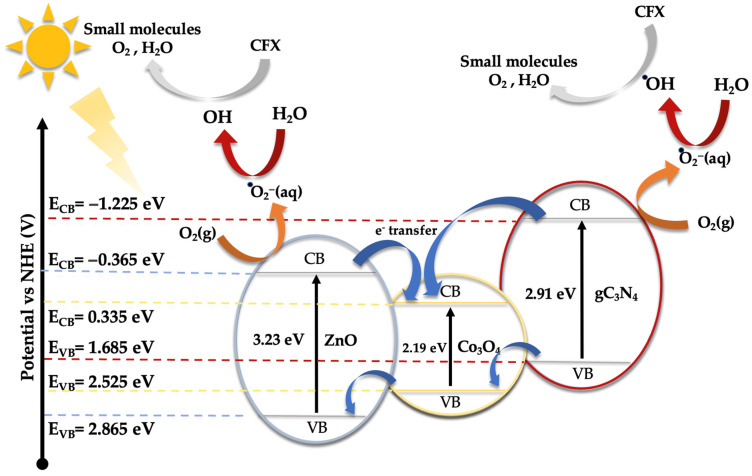
Schematic diagram of the proposed photodegradation mechanism of CFX under UV and visible radiation.

As demonstrated earlier, the integration of Co_3_O_4_-gC_3_N_4_ onto the support significantly enhances the catalytic activity. However, it is evident that an excessive amount can also diminish its efficacy. The most active catalyst, specifically 5% (Co_3_O_4_-gC_3_N_4_)@ZnONPs, possesses distinct attributes that collectively influence its efficiency, including an expanded surface area and a narrower bandgap. This might offer a higher number of active sites during the photocatalytic event, thereby yielding a greater number of photogenerated electrons. This could also contribute to a diminished recombination of these generated charges. A reduction in this recombination might be potentially augmented by the presence of Co_3_O_4_-gC_3_N_4_, though the exact mechanism remains ambiguous. The shift of the bandgap toward the visible spectrum contributes significantly to its performance. To discern the photodegradation pathway, specific scavengers were introduced to the system. In this investigation, 1,4-benzoquinone (1,4-BQ), ethylene-diaminetetraacetic acid disodium salt (EDTA-Na_2_), and methanol (MetOH) were utilized to trap superoxide radicals (·O^2−^), holes (h^+^), and hydroxyl radicals (·OH), respectively, as outlined in Figure S7. Notably, 1,4-BQ considerably impeded photoactivity, implying that the ·O^2−^ species predominantly drive the photodegradation. The introduction of EDTA had a negligible impact on photodegradation, while MetOH influenced the process significantly, though to a lesser extent than 1,4-BQ, underscoring the minimal involvement of h^+^ in the degradation. Considering these findings and the established bandgaps (refer to Figure 5), a degradation pathway involving 5% (Co_3_O_4_-gC_3_N_4_)@ZnONPs has been suggested (see Figure 8). The application of the Mulliken electronegativity principle, as delineated in references [41,42] facilitated the determination of the band edge alignment of the various constituents within the catalyst. This methodology is instrumental in elucidating the pathways of photogenerated charge carriers across the composite material. The foundational equations employed for this analysis are presented below (see Equations (1) and (2)).

E_CB_ = X − E_C_ − 0.5E_g_ (1)

E_VB_ = E_CB_ + E_g_ (2)

In the given equations, E_CB_ and E_VB_ denote the potential edges of the conduction band (CB) and valence band (VB), respectively. The term X denotes the absolute electronegativity of the material, which is a measure of its ability to attract electrons within a chemical bond. The variable E_C_ refers to the energy of free electrons relative to the hydrogen scale, which is conventionally accepted to be 4.50 eV [43]. This scale provides a standardized reference for comparing electron affinity across different materials. For the components of the catalyst under study, namely zinc oxide (ZnO), cobalt oxide (Co_3_O_4_), and graphitic carbon nitride (gC_3_N_4_), the absolute electronegativity values (X) have been cited from the literature as 5.75 [44], 5.93 [45], and 4.73 eV [46], respectively. Utilizing these electronegativity values alongside the bandgap energy (Eg) of Co_3_O_4_, which is 2.19 eV [47], allows for the calculation of the band edge positions for each component. For ZnO, the conduction band edge (E_CB_) and valence band edge (E_VB_) are calculated to be −0.365 eV and 2.865 eV, respectively. In the case of Co_3_O_4_, these values are determined to be 0.335 eV for the E_CB_ and 2.525 eV for the E_VB_. Meanwhile, for gC_3_N_4_, the computed E_CB_ and E_VB_ positions are −1.225 eV and 1.685 eV, respectively.

Under visible light exposure (see Figure 8), electrons from the valence band (VB) of gC_3_N_4_ and Co_3_O_4_ are excited to their respective conduction bands (CB), resulting in the generation of positive holes (h^+^). Because of the existing potential difference, electrons from the CB of gC_3_N_4_ can migrate to the CB of ZnONPs and Co_3_O_4_, potentially reacting with water to produce hydroxyl radicals. However, based on evaluations with varied scavengers (see Figure S7), the superoxide radical (·O^2−^) primarily facilitates the photodegradation of CFX, yielding smaller reaction by-products like CO_2_ and water. The h^+^ in the VB of gC_3_N_4_ and Co_3_O_4_ can also instigate the oxidation of CFX, contributing to its degradation. Yet, this route, as illustrated in Figure S7, is not the predominant one.

Under ultraviolet radiation, electrons in the VB of ZnONPs can be promoted to the CB, generating h^+^ in the VB. The electrons in the CB might then transfer to the CB of Co_3_O_4_, potentially reacting with water molecules to generate superoxide radicals. As observed earlier, these radicals play a pivotal role in CFX degradation. To glean deeper insights into the potential mechanism, a photoluminescence study of ZnONPs, gC_3_N_4_@ZnONPs, and the most active catalyst, namely 5% (Co_3_O_4_-gC_3_N_4_)@ZnONPs (Figure S8), was conducted. Evidently, the electron–hole recombination in gC_3_N_4_@ZnONPs is considerably attenuated compared to that in ZnONPs, and this effect is even more pronounced in the catalyst. This unambiguously suggests that the integration of Co_3_O_4_-gC_3_N_4_ deters the undesirable recombination often seen in photocatalysis, accounting for the catalyst’s outstanding performance in CFX photodegradation.

The photodegradation results of CFX achieved with the catalysts described in this study, particularly with the one exhibiting the highest activity (5% (Co_3_O_4_-gC_3_N_4_)@ZnONPs), are notably significant due to the high efficiency achieved (99%) and the relatively short time frame in which this outcome was reached. To provide context and compare with results from other catalysts also based on ZnO, the following table (Table 1) is presented, highlighting previously published results alongside those obtained in our research.

### 3.4. Photocatalytic Hydrogen Production

Before advancing to the characterization of these catalysts in the hydrogen production reaction from water, and as was previously conducted for the study of CFX catalytic photodegradation, an investigation into the optimal process conditions was carried out. For this purpose, the catalyst that demonstrated the highest efficiency in photodegradation, namely 5% (Co_3_O_4_-gC_3_N_4_)@ZnONPs, was used. The effect of the medium’s pH (Figure S9) and the catalyst loading (Figure S10) was assessed. It was observed that the optimal pH was determined to be pH = 7, while the catalyst loading was set at 60 mg/100 mL. Additionally, control studies were performed (Figure S11) to determine whether the process could proceed without light, meaning to discern if the process is purely catalytic or if it requires irradiation. As shown in Figure S11, the hydrogen production mechanism is predominantly photocatalytic. However, in the absence of light, a minor hydrogen yield of approximately 32 mmol/hg was detected, which is attributed solely to catalysis.

**Figure 9:**
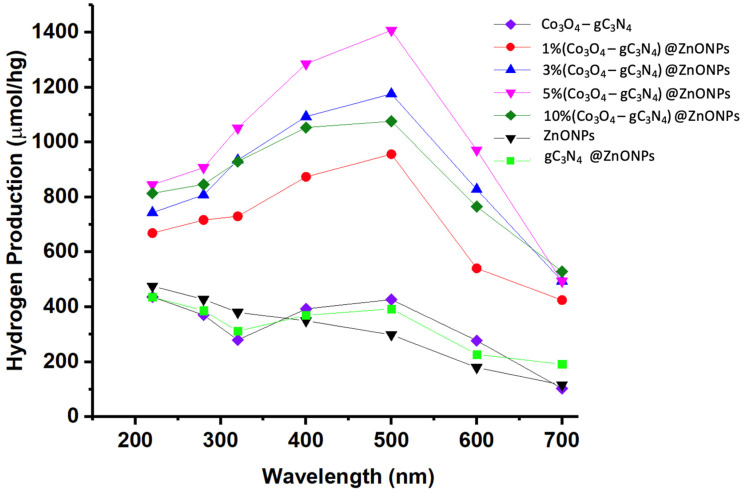
H_2_ production profiles of the synthesized materials under irradiation at different wavelengths.

Figure 9 displays the outcomes for hydrogen generation through photocatalysis across the different studied materials. The efficacy of photocatalysis was gauged using Na_2_SO_3_ (0.02 M) and Na_2_S (0.4 M) as sacrificial reagents. Activities were observed under diverse irradiation wavelengths at 220, 280, 320, 400, 500, 600, and 700 nm for all samples.

As illustrated in Figure 9, the base material of the catalyst (ZnONPs) exhibits limited activity in the visible region, though there is a slight increase as we move toward the UV range. For comparative purposes, a catalyst consisting of 5%gC_3_N_4_ on ZnONPs was prepared, and the Co_3_O_4_-gC_3_N_4_ adduct was also analyzed. In the former scenario, the inclusion of gC_3_N_4_ notably enhanced the efficiency, showcasing peak hydrogen production activity around 500 nm, distinctly in the visible region. The adduct’s activity was even more pronounced, also demonstrating peak efficiency at 500 nm. Incorporating varying proportions of Co_3_O_4_-gC_3_N_4_ onto ZnONPs resulted in the optimal hydrogen production outcomes, with all scenarios revealing peak efficiency upon irradiation at approximately 500 nm. Incremental amounts of Co_3_O_4_-gC_3_N_4_ unequivocally augmented the process, though the activity of the 10% (Co_3_O_4_-gC_3_N_4_)@ZnONPs catalyst was significantly diminished. The highest efficiency was noted with the 5% (Co_3_O_4_-gC_3_N_4_)@ZnONPs catalyst, with its hydrogen production under 500 nm irradiation determined to be 1407 mol/hg, being a value nearly five times greater than that of the base ZnONPs catalyst at the same wavelength. The specific values measured for each of the catalytic systems used, as a function of the irradiation wavelength, are presented in Table S3. Interestingly, the formed heterostructures are capable of harnessing radiation in the visible range, in contrast to the base catalyst, which demonstrates its peak activity in the UV range.

**Figure 10:**
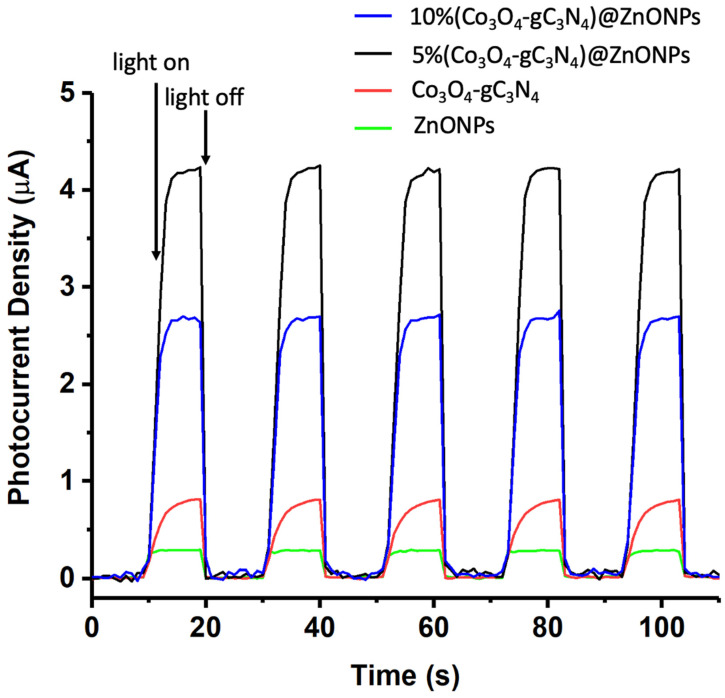
Transient photocurrent response in the light on−off processes of ZnONPs, Co_3_O_4_-gC_3_N_4_, 5% (Co_3_O_4_-gC_3_N_4_)@ZnONPs, and 10% (Co_3_O_4_-gC_3_N_4_)@ZnONPs at 500 nm.

The photoelectrochemical behaviors of the various catalysts were analyzed using a CHI660D electrochemical system in a 0.1 mol/L Na_2_SO_4_ solution. Initially, 25 mg of the nanomaterial (ZnONPs, Co_3_O_4_-gC_3_N_4_, or 5% (Co_3_O_4_-gC_3_N_4_)@ZnONPs) was dispersed in 3 mL of ethanol combined with 10 μL of a 5 wt% Nafion solution. Subsequently, 200 μL of this mixture was applied to a 1 × 1 cm^2^ FTO (fluorine-doped tin oxide) conductive glass, which functioned as the working electrode. The system employed a saturated calomel electrode (SCE) as the reference and a 1 × 1 cm^2^ platinum sheet as the counter electrode. The transient photocurrent measurements were conducted at a potential of 0.5 V (see Figure 10). Upon analysis, the fleeting photocurrent behaviors of specimens when subjected to cyclical light interruption at 500 nm indicate that the primary material (ZnONPs) has a notably lesser photocurrent density compared to the Co_3_O_4_-gC_3_N_4_ complex. Nevertheless, when this complex is integrated with ZnONPs, a significant enhancement in the photocurrent is observed, amounting to almost 16 times the value exhibited by the ZnONPs alone. This underscores that the incorporation of Co_3_O_4_-gC_3_N_4_ can efficaciously expedite the dispersion of photo-induced carriers due to the creation of heterojunctions. An over-represented concentration of Co_3_O_4_-gC_3_N_4_, as exemplified in the catalyst 10% (Co_3_O_4_-gC_3_N_4_)@ZnONPs, curtails the material’s photocurrent, as illustrated in Figure 10, which is manifestly counterproductive for the optimal separation of the photogenerated carriers. The data presented in Figure 10 are particularly elucidative as they validate the H_2_ generation values outlined in Figure 9.

To complement the results presented earlier and to delve deeper into the effect of the photogenerated carriers during the H_2_ generation process, an additional study was conducted using a hole (h^+^) scavenger, namely EDTA-Na_2_. As illustrated in Figure S12, the addition of EDTA-Na_2_ to the reaction mixture resulted in an increase in hydrogen production for all catalysts tested and under irradiation at all wavelengths examined. Based on the results presented earlier, the catalysts studied in this research already demonstrate efficient electron–hole separation. However, the observed behavior upon adding EDTA-Na_2_ can be rationalized by the further intensified reduction of the electron–hole recombination process, leading to an enhanced H_2_ production.

Finally, a recyclability study of the most active catalyst, specifically 5% (Co_3_O_4_-gC_3_N_4_)@ZnONPs, was conducted. For this purpose, the catalyst was subjected to 10 usage cycles. After each cycle, the catalyst was recovered from the reaction mixture via centrifugation (3000 rpm, 15 min) and subsequently washed through two centrifugation–washing cycles using water. The catalyst was then dried at 60 °C for 3 h before the next usage cycle. The obtained results are presented in Figure S13. As observed, a decline in hydrogen production is evident from the first cycle and persists throughout the 10 studied cycles. After the final cycle, the measured H_2_ production was 979 mol/hg, indicating an efficiency drop of approximately 30%. This result could be attributed to potential leaching of Co_3_O_4_ or even the loss of Co_3_O_4_-gC_3_N_4_ during the catalyst’s usage and regeneration processes. To substantiate this observation, a quantitative analysis of Co was performed using XPS (not shown). The initial elemental composition of the catalyst (5% (Co_3_O_4_-gC_3_N_4_)@ZnONPs) was as follows: cobalt 3%, carbon 15%, zinc 31%, and oxygen 51%. After the 10th cycle, the composition shifted to the following: cobalt 1%, carbon 17%, zinc 26%, and oxygen 56%. Consequently, the Co/Zn ratio decreased from approximately 0.097 to 0.038, indicating cobalt leaching. This aligns with observations made during the photodegradation of CFX, suggesting a loss of Co_3_O_4_ from the heterostructure during the catalyst’s operation and subsequent reactivation. Given the nature of the Co_3_O_4_-gC_3_N_4_ hybrids, obtained through thermal treatment from urea and cobalt(II) acetate, Co_3_O_4_ particles are likely distributed throughout the graphitic carbon nitride. Consequently, Co_3_O_4_ leaching might occur alongside the loss of gC_3_N_4_, though this possibility will require further evaluation in subsequent studies.

**Figure 11:**
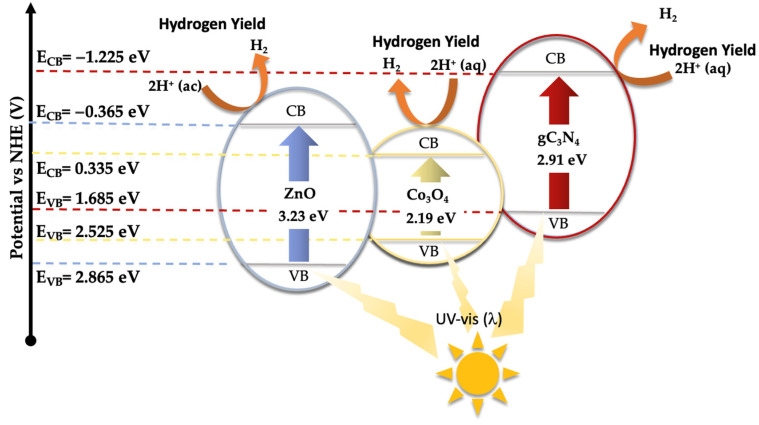
Schematic mechanism proposed for the hydrogen production using the 5% (Co_3_O_4_-gC_3_N_4_)@ZnONPs catalyst under UV–visible irradiation.

Based on the previously presented results, a potential mechanism for the catalyst’s role in the photocatalytic hydrogen production through water splitting has been proposed (see Figure 11). According to this mechanism, by incorporating Co_3_O_4_-gC_3_N_4_ onto the ZnONPs support, the catalyst can absorb in the visible spectrum region, significantly enhancing the material’s practical application. The photogenerated electrons serve to reduce H^+^ to H_2_, which also counteracts the electron–hole recombination process. However, as indicated in Figure S12, when EDTA-Na_2_, a hole scavenger, is added to the reaction medium, an increase in the H_2_ production rate is observed. This suggests that under standard reaction conditions, a minor fraction of electrons is not utilized for H_2_ production but is swiftly returned to the VB where they recombine with the holes.

To contextualize the findings of our study, we have juxtaposed our results against those previously published in the domain. To the best of our knowledge, there are no reported outcomes using catalysts akin to those developed in this study, specifically utilized for concurrent photodegradation and hydrogen production. Consequently, our research pioneers in this territory, addressing two distinctly critical objectives, namely energy sustainability and environmental conservation.

Previously published studies have primarily focused on ZnO-based catalysts, particularly in the photodegradation of pollutants such as CFX. Notably, Yang et al. [52] achieved approximately 97% CFX photodegradation within 48 min using ZnO-Ag_2_O/porous gC_3_N_4_ composites. Similarly, Wang et al. [53] reported over 87% degradation of CFX within 120 min using ZnO-Ag-Ag_3_PO_4_ composites. In another significant study, Swaminathan et al. [54] documented over 98% CFX photodegradation in 1 h with a ternary system based on rGO, BiVO_4_, and ZnO. Our research group has recently observed analogous results employing Au@ZnONPs-MoS_2_-rGO nanocomposites [5], with a 96% photodegradation rate of CFX in 1 h. The simultaneous application of catalysts for both decontamination processes and hydrogen production is markedly underrepresented in the existing literature. However, the results of our current investigation, achieving hydrogen production rates exceeding 1400 mmol/hg, are decidedly noteworthy within this context. For instance, Liang et al. [55] reported hydrogen productions of 1068 mol/g over 4 h using TiO_2_–ZnO/Au catalysts under visible light irradiation. In a more recent study, Ahmad et al. [56] noted exceptional hydrogen production values (4655 mmol/hg) for ZnO–CuO–Au composites in the presence of glycerol. Employing more intricate heterostructures like those developed by Xie et al. [57], based on ZnIn_2_S_4_/ZnO under full-spectrum irradiation, yielded values as high as 13,638 μmol/hg (in water–ethanol mixtures) and 3036 μmol/hg (in water). Vattikuti et al. [58] also presented compelling results with SnO_2_−ZnO quantum dots anchored on gC_3_N_4_ nanosheets, achieving hydrogen productions of 13,673 μmol/g over 5 h, while actively photodegrading rhodamine B, with degradation rates nearing 99% in 1 h under UV–vis irradiation.

These findings collectively signify the substantial progress made in recent years and the clear advancements in these types of processes. Despite this, the scientific community remains distanced from the utilization of universal catalysts that perform with maximal efficiency across diverse applications and practical implementations. Nonetheless, the continual emergence of these advancements progressively propels us closer to that eventuality.

## 4. Conclusions

In this study, we assessed the photocatalytic capabilities of several catalysts, including ZnONPs and Co_3_O_4_-gC_3_N_4_, focusing on their efficiency in degrading ciprofloxacin (CFX) and producing hydrogen (H_2_) through water splitting. Our experiments revealed that CFX underwent rapid photodegradation within 60 min, with degradation rates varying between approximately 80% and 99%. The catalyst that emerged as the most effective was the 5% (Co_3_O_4_-gC_3_N_4_)@ZnONPs. The by-products resulting from the photodegradation of CFX were identified through gas chromatography–mass spectrometry (GC-MS) analysis. Additionally, photoluminescence studies, alongside bandgap evaluations and the assessment of the influence of various scavengers on the reaction environment, shed light on a photodegradation mechanism for CFX that predominantly involves the generation of superoxide radicals.

The efficacy of these catalysts was also tested for photocatalytic hydrogen production via water splitting, where they showed promising activity. Particularly, these materials achieved significant hydrogen yields under visible light (500 nm), with the most efficient catalyst reaching a production rate of 1407 μmol/hg. Recyclability tests for both the photocatalytic degradation of CFX and H_2_ production revealed varying behaviors among the catalysts. After 15 cycles, a 6% decrease in activity was observed for CFX degradation, whereas H_2_ production saw a more substantial reduction of 30%. This decline in catalytic performance was attributed to the loss of cobalt, a critical component influencing the catalyst’s effectiveness. Although cobalt loss was noted in both processes, its detrimental effect on efficiency was more pronounced in H_2_ production than in CFX degradation, an observation that merits further exploration.

In summary, this research underscores the versatility and potential of these catalytic materials for environmental and energy applications. Specifically, it demonstrates their capability in addressing waterborne organic pollutants and in contributing to energy generation efforts. The findings not only advance our understanding of photocatalytic processes but also point toward the sustainable use of such catalysts in mitigating environmental pollution and harnessing renewable energy sources.

## Figures and Tables

**Table 1:** Recent results obtained with hybrid catalysts in the CFX photodegradation process.

Reference	Catalyst	Efficiency (%)	Time (min)
[48]	TiO_2_-ZnO@Oxygen-doped gC_3_N_4_	99.7	60
[49]	ZnO/g-C_3_N_4_	93.8	60
[50]	g-C_3_N_4_/ZnO	96	180
[51]	S-C_3_N_4_/ZnO-Chitosan	93 (UV) 69 (Vis)	60
[52]	ZnO-Ag_2_O/g-C_3_N_4_	97.4	48
This work	5% (Co_3_O_4_-gC_3_N_4_)@ZnONPs	99	60

## Data Availability

The data is contained in the article and is available from the corresponding authors on reasonable request.

## Supplementary Materials

The following supporting information can be downloaded at: https://www.mdpi.com/article/10.3390/ma17051059/s1. Figure S1. Photocatalytic activity of 5% (Co_3_O_4_-gC_3_N_4_)@ZnONPs on the photodegradation of CFX under irradiation at different pH values; Figure S2. Evaluation of the initial concentration of CFX on the catalytic efficiency of 5% (Co_3_O_4_-gC_3_N_4_)@ZnONPs (a), and 10% (Co_3_O_4_-gC_3_N_4_)@ZnONPs (b), in the photodegradation reaction; Figure S3. Evaluation of the initial concentration of 5% (Co_3_O_4_-gC_3_N_4_)@ZnONPs (a), and 10% (Co_3_O_4_-gC_3_N_4_)@ZnONPs (b) on the efficiency of the photodegradation reaction of CFX. Figure S4. First-order kinetic plots of −ln(C/C_0_) versus irradiation time, using different catalysts; Figure S5. Control experiments for 5% (Co_3_O_4_-gC_3_N_4_)@ZnONPs with CFX, under irradiation; Figure S6. Recyclability of 5% (Co_3_O_4_-gC_3_N_4_)@ZnONPs after 15 consecutive catalytic cycles of photodegradation of CFX under irradiation; Figure S7. Photodegradation of CFX by 5% (Co_3_O_4_-gC_3_N_4_)@ZnONPs in the presence of various scavengers; Figure S8. PL spectra of ZnONPs (a), gC_3_N_4_@ZnONPs (b), and 5% (Co_3_O_4_-gC_3_N_4_)@ZnONPs (c); Figure S9. Effect of pH on the photocatalytic activity of the 5% (Co_3_O_4_-gC_3_N_4_)@ZnONPs catalyst for hydrogen production; Figure S10. Evaluation of the initial concentration of 5% (Co_3_O_4_-gC_3_N_4_)@ZnONPs (a), on the efficiency of hydrogen production; Figure S11. Control experiments for 5% (Co_3_O_4_-gC_3_N_4_)@ZnONPs on the efficiency of hydrogen production; Figure S12. Hydrogen production via water splitting using various catalysts under irradiation, and also in the presence of a hole scavenger, namely EDTA-Na_2_; Figure S13. Recyclability of 5% (Co_3_O_4_-gC_3_N_4_)@ZnONPs after 10 consecutive catalytic cycles of hydrogen production, under irradiation at 500 nm; Table S1. BET surface area of the as-synthesized materials; Table S2. The pseudo-first-order kinetics constants for the photodegradation of CFX using the as-synthesized catalysts; Table S3. Maximum H_2_ production (µmol/hg) by the different catalysts and precursors, under irradiation at several wavelengths. All values obtained are affected by 5% error.

## References

[1] Perera F., Nadeau K. (2022). Climate Change, Fossil-Fuel Pollution, and Children’s Health. N. Engl. J. Med..

[2] Lelieveld J., Klingmüller K., Pozzer A., Burnett R.T., Haines A., Ramanathan V. (2019). Effects of Fossil Fuel and Total Anthropogenic Emission Removal on Public Health and Climate. Proc. Natl. Acad. Sci. USA.

[3] Perera F. (2017). Pollution from Fossil-Fuel Combustion Is the Leading Environmental Threat to Global Pediatric Health and Equity: Solutions Exist. Int. J. Environ. Res. Public Health.

[4] Machín A., Soto-Vázquez L., Colón-Cruz C., Valentín-Cruz C.A., Claudio-Serrano G.J., Fontánez K., Resto E., Petrescu F.I., Morant C., Márquez F. (2021). Photocatalytic Activity of Silver-Based Biomimetics Composites. Biomimetics.

[5] Machín A., Soto-Vázquez L., García D., Cotto M.C., Ortiz D., Berríos-Rolón P.J., Fontánez K., Resto E., Morant C., Petrescu F. (2023). Photodegradation of Ciprofloxacin and Levofloxacin by Au@ZnONPs-MoS_2_-rGO Nanocomposites. Catalysts.

[6] Serwecińska L. (2020). Antimicrobials and Antibiotic-Resistant Bacteria: A Risk to the Environment and to Public Health. Water.

[7] Machín A., Cotto M., Duconge J., Arango J.C., Morant C., Pinilla S., Soto-Vázquez L., Resto E., Márquez F. (2018). Hydrogen Production via Water Splitting Using Different Au@ZnO Catalysts under UV–Vis Irradiation. J. Photochem. Photobiol. A Chem..

[8] Fontánez K., García D., Ortiz D., Sampayo P., Hernández L., Cotto M., Ducongé J., Díaz F., Morant C., Petrescu F. (2022). Biomimetic Catalysts Based on Au@TiO_2_-MoS_2_-CeO_2_ Composites for the Production of Hydrogen by Water Splitting. Int. J. Mol. Sci..

[9] Machín A., Fontánez K., García D., Sampayo P., Colón-Cruz C., Claudio-Serrano G.J., Soto-Vázquez L., Resto E., Petrescu F.I., Morant C. (2022). Hydrogen Production and Degradation of Ciprofloxacin by Ag@TiO_2_-MoS_2_ Photocatalysts. Catalysts.

[10] Machín A., Fontánez K., Duconge J., Cotto M.C., Petrescu F.I., Morant C., Márquez F. (2022). Photocatalytic Degradation of Fluoroquinolone Antibiotics in Solution by Au@ZnO-rGO-gC_3_N_4_ Composites. Catalysts.

[11] Machín A., Arango J.C., Fontánez K., Cotto M., Duconge J., Soto-Vázquez L., Resto E., Petrescu F.I.T., Morant C., Márquez F. (2020). Biomimetic Catalysts Based on Au@ZnO–Graphene Composites for the Generation of Hydrogen by Water Splitting. Biomimetics.

[12] Ismael M. (2020). The Photocatalytic Performance of the ZnO/g-C_3_N_4_ Composite Photocatalyst toward Degradation of Organic Pollutants and Its Inactivity toward Hydrogen Evolution: The Influence of Light Irradiation and Charge Transfer. Chem. Phys. Lett..

[13] Long X., Li F., Gao L., Hu Y., Hu H., Jin J., Ma J. (2018). Heterojunction and Oxygen Vacancy Modification of ZnO Nanorod Array Photoanode for Enhanced Photoelectrochemical Water Splitting. ChemSusChem.

[14] Rini N.P., Istiqomah N.I., Suharyadi E. (2023). Photocatalytic Activity of CoO/ZnO Nanocrystalline for Dye Wastewater Treatment under UV Light. MSF.

[15] Zada A., Khan M., Hussain Z., Shah M.I.A., Ateeq M., Ullah M., Ali N., Shaheen S., Yasmeen H., Ali Shah S.N. (2022). Extended Visible Light Driven Photocatalytic Hydrogen Generation by Electron Induction from G-C_3_N_4_ Nanosheets to ZnO through the Proper Heterojunction. Z. Für Phys. Chem..

[16] Thi Viet Ha T., Minh Viet N., Le Minh Tri N. (2022). Study on Synthesis and Structural Characterization of ZnO-Doped g-C_3_N_4_ Materials for Treatment of Ciprofloxacin Antibiotic in Water. Proceedings of the 2022 7th National Scientific Conference on Applying New Technology in Green Buildings (ATiGB).

[17] Li K., Chen M., Chen L., Zhao S., Xue W., Han Z., Han Y. (2023). Synthesis of G-C_3_N_4_ Derived from Different Precursors for Photodegradation of Sulfamethazine under Visible Light. Processes.

[18] Suhag M.H., Khatun A., Tateishi I., Furukawa M., Katsumata H., Kaneco S. (2023). One-Step Fabrication of the ZnO/g-C_3_N_4_ Composite for Visible Light-Responsive Photocatalytic Degradation of Bisphenol E in Aqueous Solution. ACS Omega.

[19] Makuła P., Pacia M., Macyk W. (2018). How to Correctly Determine the Band Gap Energy of Modified Semiconductor Photocatalysts Based on UV–Vis Spectra. J. Phys. Chem. Lett..

[20] Cao D., Gong S., Shu X., Zhu D., Liang S. (2019). Preparation of ZnO Nanoparticles with High Dispersibility Based on Oriented Attachment (OA) Process. Nanoscale Res. Lett..

[21] Cao S.-W., Yuan Y.-P., Fang J., Shahjamali M.M., Boey F.Y.C., Barber J., Joachim Loo S.C., Xue C. (2013). In-Situ Growth of CdS Quantum Dots on g-C_3_N_4_ Nanosheets for Highly Efficient Photocatalytic Hydrogen Generation under Visible Light Irradiation. Int. J. Hydrog. Energy.

[22] Morant C., Andrey J., Prieto P., Mendiola D., Sanz J.M., Elizalde E. (2006). XPS Characterization of Nitrogen-doped Carbon Nanotubes. Phys. Status Solidi (A).

[23] Singh P., Simanjuntak F.M., Wu Y.-C., Kumar A., Zan H.-W., Tseng T.-Y. (2020). Sensing Performance of Gas Sensors Fabricated from Controllably Grown ZnO-Based Nanorods on Seed Layers. J. Mater Sci..

[24] Santra B., Pal S., Saha S., Kanjilal A. (2023). Tailoring Structural, Chemical, and Photocatalytic Properties of ZnO@β-SiC Composites: The Effect of Annealing Temperature and Environment. ACS Omega.

[25] Kim K.-H., Choi Y.-H. (2022). Surface Oxidation of Cobalt Carbonate and Oxide Nanowires by Electrocatalytic Oxygen Evolution Reaction in Alkaline Solution. Mater. Res. Express.

[26] Xiang Q., Yu J., Jaroniec M. (2011). Preparation and Enhanced Visible-Light Photocatalytic H_2_ -Production Activity of Graphene/C_3_ N_4_ Composites. J. Phys. Chem. C.

[27] Briggs D., Seah M. (1994). Practical Surface Analysis.

[28] Naseri A., Samadi M., Mahmoodi N.M., Pourjavadi A., Mehdipour H., Moshfegh A.Z. (2017). Tuning Composition of Electrospun ZnO/CuO Nanofibers: Toward Controllable and Efficient Solar Photocatalytic Degradation of Organic Pollutants. J. Phys. Chem. C.

[29] Menezes P.W., Indra A., Gutkin V., Driess M. (2017). Boosting Electrochemical Water Oxidation through Replacement of O_h_ Co Sites in Cobalt Oxide Spinel with Manganese. Chem. Commun..

[30] Sparks T.D., Gurlo A., Bekheet M.F., Gaultois M.W., Cherkashinin G., Laversenne L., Clarke D.R. (2019). High-Temperature Structure of Co_3_O_4_: Understanding Spinel Inversion Using in Situ and Ex Situ Measurements. Phys. Rev. B.

[31] Liang H., Raitano J.M., Zhang L., Chan S.-W. (2009). Controlled Synthesis of Co_3_O_4_ Nanopolyhedrons and Nanosheets at Low Temperature. Chem. Commun..

[32] Stankovich S., Dikin D.A., Piner R.D., Kohlhaas K.A., Kleinhammes A., Jia Y., Wu Y., Nguyen S.T., Ruoff R.S. (2007). Synthesis of Graphene-Based Nanosheets via Chemical Reduction of Exfoliated Graphite Oxide. Carbon.

[33] Li J., Wang Y., Li X., Gao Q., Zhang S. (2021). A Facile Synthesis of High-Crystalline g-C_3_N_4_ Nanosheets with Closed Self-Assembly Strategy for Enhanced Photocatalytic H_2_ Evolution. J. Alloys Compd..

[34] Cuscó R., Alarcón-Lladó E., Ibáñez J., Artús L., Jiménez J., Wang B., Callahan M.J. (2007). Temperature Dependence of Raman Scattering in ZnO. Phys. Rev. B.

[35] Mottola S., Mancuso A., Sacco O., Vaiano V., De Marco I. (2023). Photocatalytic Systems Based on ZnO Produced by Supercritical Antisolvent for Ceftriaxone Degradation. Catalysts.

[36] Na C.W., Woo H.-S., Kim H.-J., Jeong U., Chung J.-H., Lee J.-H. (2012). Controlled Transformation of ZnO Nanobelts into CoO/Co_3_O_4_ Nanowires. CrystEngComm.

[37] Gwag J.S., Sohn Y.-K. (2012). Interfacial Natures and Controlling Morphology of Co Oxide Nanocrystal Structures by Adding Spectator Ni Ions. Bull. Korean Chem. Soc..

[38] Zhao C., Li Y., Chu H., Pan X., Ling L., Wang P., Fu H., Wang C.-C., Wang Z. (2021). Construction of Direct Z-Scheme Bi_5_O_7_I/UiO-66-NH_2_ Heterojunction Photocatalysts for Enhanced Degradation of Ciprofloxacin: Mechanism Insight, Pathway Analysis and Toxicity Evaluation. J. Hazard. Mater..

[39] Shen C.-H., Wen X.-J., Fei Z.-H., Liu Z.-T., Mu Q.-M. (2020). Visible-Light-Driven Activation of Peroxymonosulfate for Accelerating Ciprofloxacin Degradation Using CeO2/Co_3_O_4_ p-n Heterojunction Photocatalysts. Chem. Eng. J..

[40] Dang V.D., Adorna J., Annadurai T., Bui T.A.N., Tran H.L., Lin L.-Y., Doong R.-A. (2021). Indirect Z-Scheme Nitrogen-Doped Carbon Dot Decorated Bi_2_MoO_6_/g-C_3_N_4_ Photocatalyst for Enhanced Visible-Light-Driven Degradation of Ciprofloxacin. Chem. Eng. J..

[41] Jourshabani M., Shariatinia Z., Badiei A. (2018). Synthesis and Characterization of Novel Sm_2_O_3_/S-Doped g-C_3_N_4_ Nanocomposites with Enhanced Photocatalytic Activities under Visible Light Irradiation. Appl. Surf. Sci..

[42] Prabavathi S.L., Saravanakumar K., Nkambule T.T.I., Muthuraj V., Mamba G. (2020). Enhanced Photoactivity of Cerium Tungstate-Modified Graphitic Carbon Nitride Heterojunction Photocatalyst for the Photodegradation of Moxifloxacin. J. Mater Sci. Mater Electron..

[43] Cao J., Li X., Lin H., Chen S., Fu X. (2012). In Situ Preparation of Novel p–n Junction Photocatalyst BiOI/(BiO)_2_CO_3_ with Enhanced Visible Light Photocatalytic Activity. J. Hazard. Mater..

[44] Chen C., Bi W., Xia Z., Yuan W., Li L. (2020). Hydrothermal Synthesis of the CuWO_4_ /ZnO Composites with Enhanced Photocatalytic Performance. ACS Omega.

[45] Bai S., Liu H., Luo R., Chen A., Li D. (2014). SnO_2_ @Co_3_ O_4_ p–n Heterostructures Fabricated by Electrospinning and Mechanism Analysis Enhanced Acetone Sensing. RSC Adv..

[46] Jiménez-Salcedo M., Monge M., Tena M.T. (2021). The Photocatalytic Degradation of Sodium Diclofenac in Different Water Matrices Using G-C3N4 Nanosheets: A Study of the Intermediate by-Products and Mechanism. J. Environ. Chem. Eng..

[47] Bhargava R., Khan S., Ahmad N., Ansari M.M.N. (2018). Investigation of Structural, Optical and Electrical Properties of Co_3_O_4_ Nanoparticles.

[48] Buu T.T., Cong C.Q., Quan V.M., Ngoc B.K., Nam N.T.H., Thao L.T.P., Tam D.H.M., Han L.G., Hieu N.H. (2023). Construction of Z-Scheme Heterojunction TiO_2_-ZnO@Oxygen-Doped gC_3_N_4_ Composite for Enhancing H_2_O_2_ Photoproduction and Removal of Pharmaceutical Pollutants under Visible Light. Surf. Interfaces.

[49] Van Thuan D., Nguyen T.B.H., Pham T.H., Kim J., Hien Chu T.T., Nguyen M.V., Nguyen K.D., Al-onazi W.A., Elshikh M.S. (2022). Photodegradation of Ciprofloxacin Antibiotic in Water by Using ZnO-Doped g-C_3_N_4_ Photocatalyst. Chemosphere.

[50] Gayathri K., Teja Y.N., Prakash R.M., Hossain M.S., Alsalme A., Sundaravadivel E., Sakar M. (2022). In Situ-Grown ZnO Particles on g-C_3_N_4_ Layers: A Direct Z-Scheme-Driven Photocatalyst for the Degradation of Dye and Pharmaceutical Pollutants under Solar Irradiation. J. Mater Sci. Mater Electron..

[51] Gupta B., Gupta A.K. (2022). Photocatalytic Performance of 3D Engineered Chitosan Hydrogels Embedded with Sulfur-Doped C_3_N_4_/ZnO Nanoparticles for Ciprofloxacin Removal: Degradation and Mechanistic Pathways. Int. J. Biol. Macromol..

[52] Rong X., Qiu F., Jiang Z., Rong J., Pan J., Zhang T., Yang D. (2016). Preparation of Ternary Combined ZnO-Ag_2_O/Porous g-C_3_N_4_ Composite Photocatalyst and Enhanced Visible-Light Photocatalytic Activity for Degradation of Ciprofloxacin. Chem. Eng. Res. Des..

[53] Du C., Song J., Tan S., Yang L., Yu G., Chen H., Zhou L., Zhang Z., Zhang Y., Su Y. (2021). Facile Synthesis of Z-Scheme ZnO/Ag/Ag_3_PO_4_ Composite Photocatalysts with Enhanced Performance for the Degradation of Ciprofloxacin. Mater. Chem. Phys..

[54] Raja A., Rajasekaran P., Selvakumar K., Arunpandian M., Kaviyarasu K., Asath Bahadur S., Swaminathan M. (2020). Visible Active Reduced Graphene Oxide-BiVO_4_-ZnO Ternary Photocatalyst for Efficient Removal of Ciprofloxacin. Sep. Purif. Technol..

[55] Liang Y., Li W., Wang X., Zhou R., Ding H. (2022). TiO_2_–ZnO/Au Ternary Heterojunction Nanocomposite: Excellent Antibacterial Property and Visible-Light Photocatalytic Hydrogen Production Efficiency. Ceram. Int..

[56] Ahmad I., Shukrullah S., Naz M.Y., Bhatti H.N., Khalid N.R., Ullah S. (2023). Rational Design of ZnO–CuO–Au S-Scheme Heterojunctions for Photocatalytic Hydrogen Production under Visible Light. Int. J. Hydrog. Energy.

[57] Xie Z., Xie L., Qi F., Liu H., Meng L., Wang J., Xie Y., Chen J., Lu C.-Z. (2023). Efficient Photocatalytic Hydrogen Production by Space Separation of Photo-Generated Charges from S-Scheme ZnIn_2_S_4_/ZnO Heterojunction. J. Colloid Interface Sci..

[58] Vattikuti S.V.P., Reddy P.A.K., Shim J., Byon C. (2018). Visible-Light-Driven Photocatalytic Activity of SnO_2_ –ZnO Quantum Dots Anchored on g-C_3_N_4_ Nanosheets for Photocatalytic Pollutant Degradation and H_2_ Production. ACS Omega.

