# Supplementary material for: Synergistic Effects of Co3O4-gC3N4-Coated ZnO Nanoparticles: A Novel Approach for Enhanced Photocatalytic Degradation of Ciprofloxacin and Hydrogen Evolution via Water Splitting
Source: Materials (Basel). 2024 Feb 25;17(5):1059. doi: 10.3390/ma17051059 (PMC10935200; doi:10.3390/ma17051059)
Supplement: Supplementary file 1 [file materials-17-01059-s001.zip › materials-2885861-SI.pdf]

materials

MDPI

Supplementary Materials

Synergistic Effects of Co_3O_4-gC_3N_4-Coated ZnO Nanoparticles: A Novel Approach for Enhanced Photocatalytic Degradation of Ciprofloxacin and Hydrogen Evolution via Water Splitting

Abniel Machín [1,*], Carmen Morant [2], Loraine Soto-Vázquez [3], Edgard Resto [3], José Ducongé [4], María Cotto [4], Pedro J. Berríos-Rolón [4], Cristian Martínez-Perales [4] and Francisco Márquez [4,*]

1 Environmental Catalysis Research Lab, Division of Science, Technology and Environment, Cupey Campus, Universidad Ana G. Méndez, Cupey, PR 00926, USA
2 Department of Applied Physics, Autonomous University of Madrid, and Instituto de Ciencia de Materiales Nicolás Cabrera, 28049 Madrid, Spain; c.morant@uam.es
3 Materials Characterization Center Inc., Molecular Sciences Research Center, University of Puerto Rico, San Juan, PR 00926, USA; loraine.soto@mcc.com.pr (L.S.-V.); restoe@mcc.com.pr (E.R.)
4 Nanomaterials Research Group, Department of Natural Sciences and Technology, Division of Natural Sciences, Technology and Environment, Universidad Ana G. Méndez-Gurabo Campus, Gurabo, PR 00778, USA; jduconge@uagm.edu (J.D.); mcotto48@uagm.edu (M.C.); berriosp1@uagm.edu (P.J.B.-R.); cmartinez372@email.uagm.edu (C.M.-P.)
* Correspondence: machina1@uagm.edu (A.M.); fmarquez@uagm.edu (F.M.); Tel.: +1-787-878-2612 (ext. 220) (A.M.); +1-787-743-7979 (ext. 4250) (F.M.)

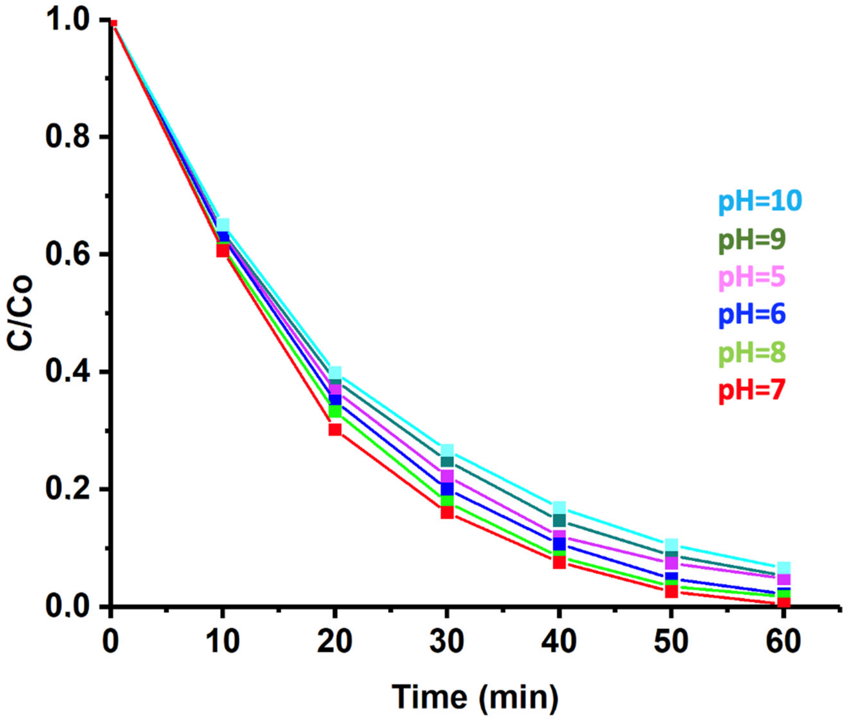

Figure S1. Photocatalytic activity of 5%(Co_3O_4-gC_3N_4)@ZnONPs on the phodegradation of CFX under irradiation at different pH.

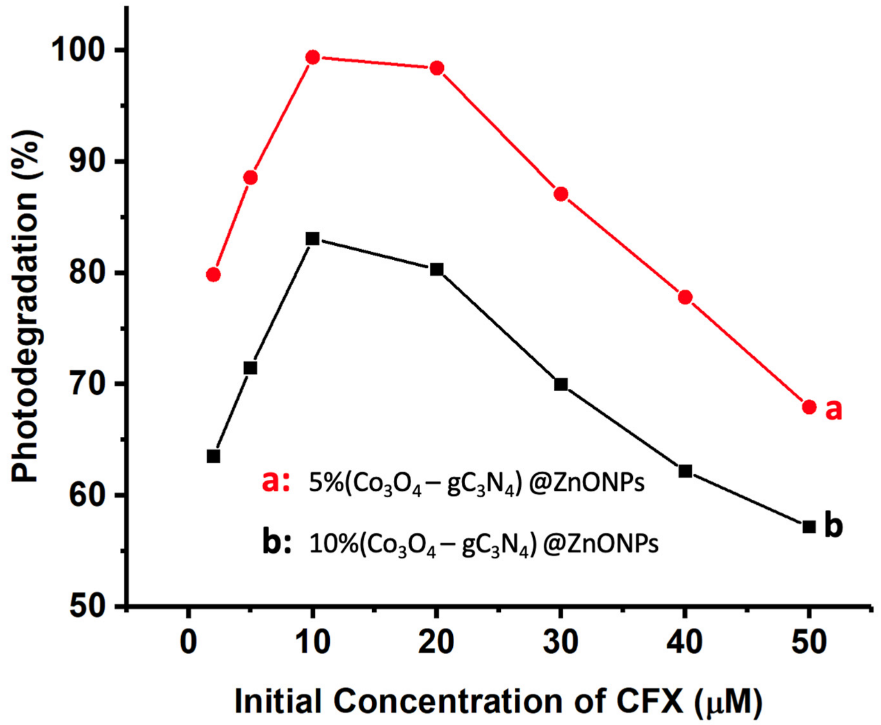

Figure S2. Photocatalytic activity of 5%(Co_3O_4-gC_3N_4)@ZnONPs on the phodegradation of CFX under irradiation at different pH.

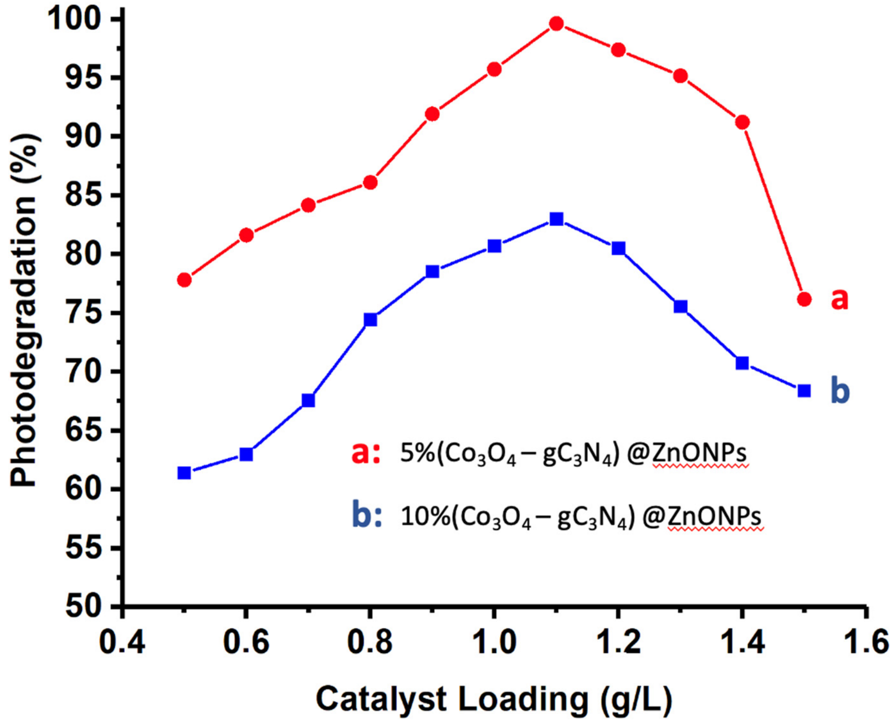

Figure S3. Evaluation of the initial concentration of 5%(Co_3O_4-gC_3N_4)@ZnONPs (**a**), and 10%(Co_3O_4-gC_3N_4)@ZnONPs (**b**) on the efficiency of the photodegradation reaction of CFX.

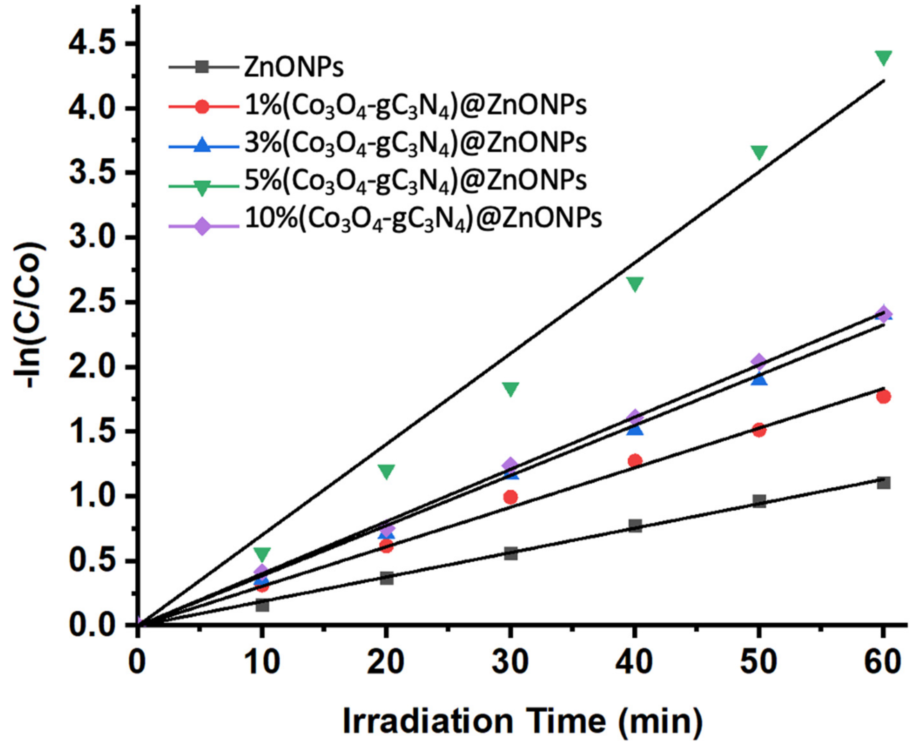

Figure S4. First-order kinetic plots of −ln(C/C_0) versus irradiation time, using different catalysts.

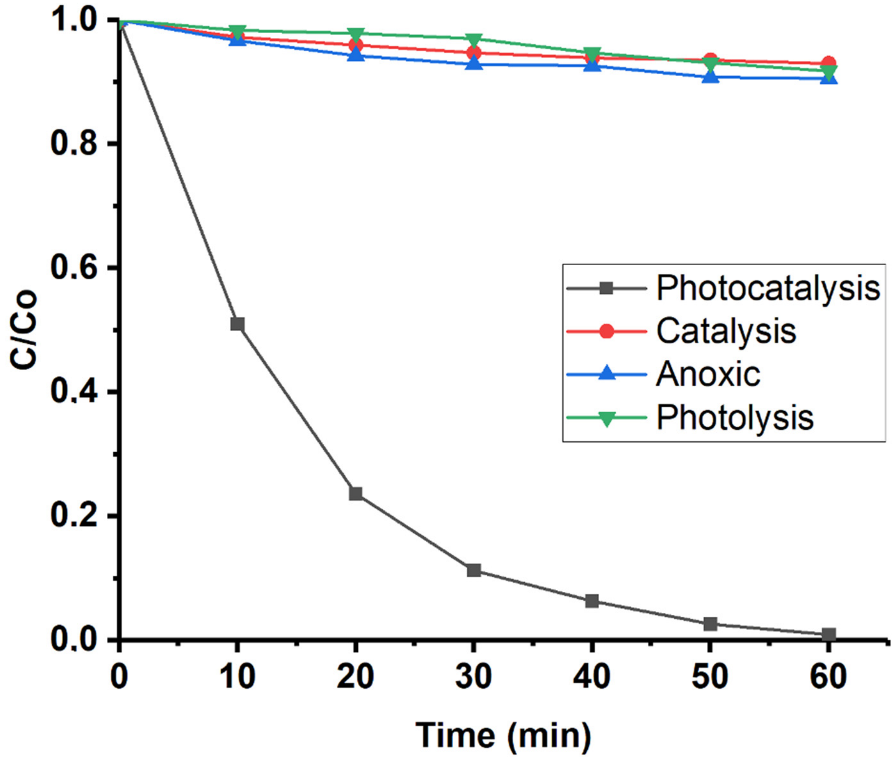

Figure S5. Control experiments for 5%(Co_3O_4-gC_3N_4)@ZnONPs with CFX, under irradiation.

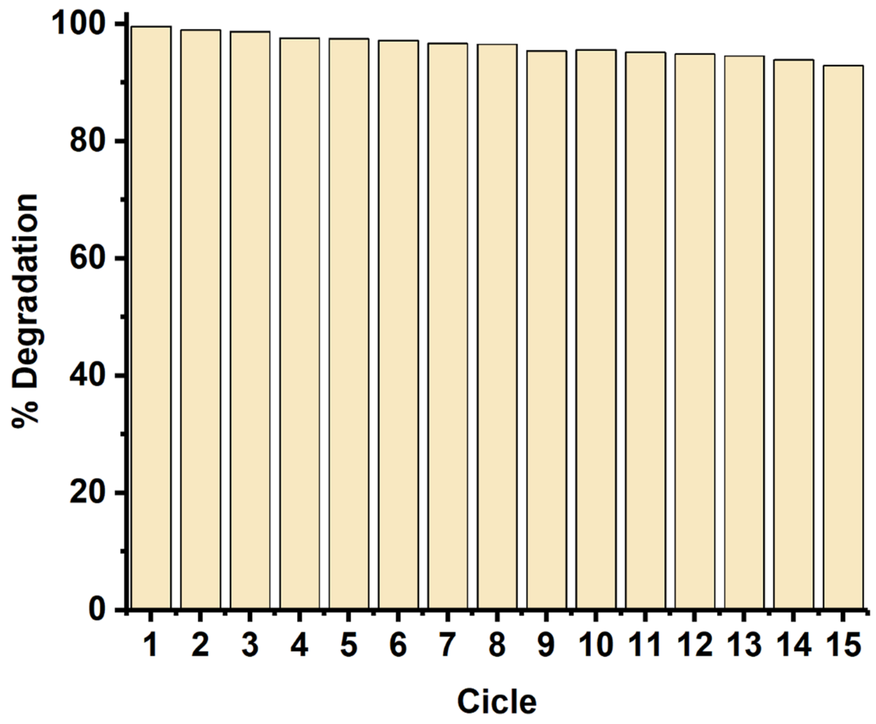

Figure S6. Recyclability of 5%(Co_3O_4-gC_3N_4)@ZnONPs after 15 consecutive catalytic cycles of photodegradation of CFX under irradiation.

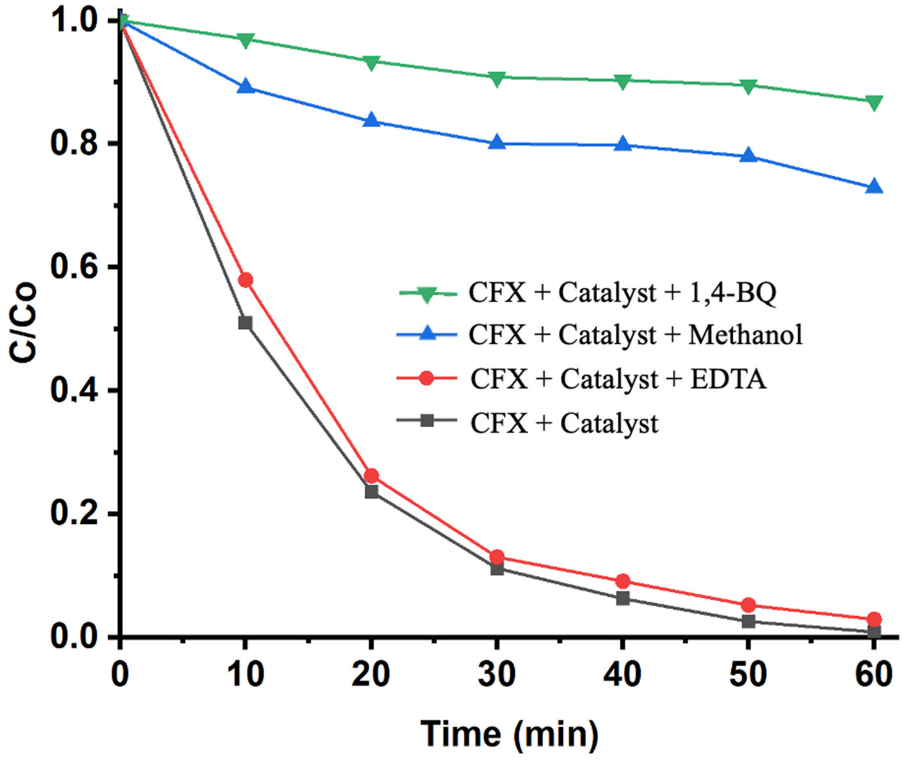

Figure S7. Photodegradation of CFX by 5%(Co_3O_4-gC_3N_4)@ZnONPs in the presence of various scavengers.

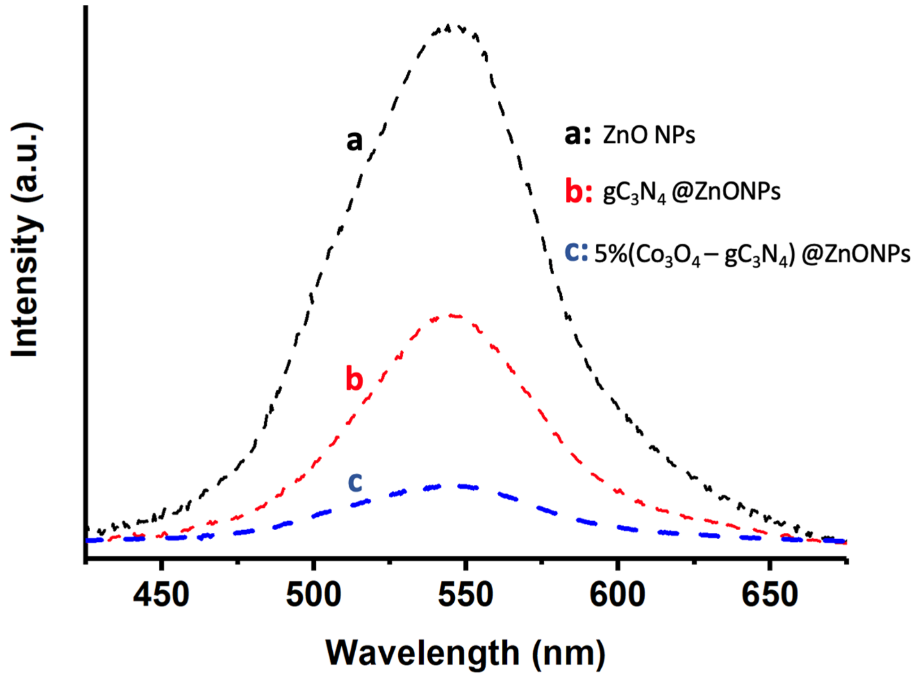

Figure S8. PL spectra of ZnONPs (**a**), gC_3N_4@ZnONPs (**b**), and 5%(Co_3O_4-gC_3N_4)@ZnONPs (**c**).

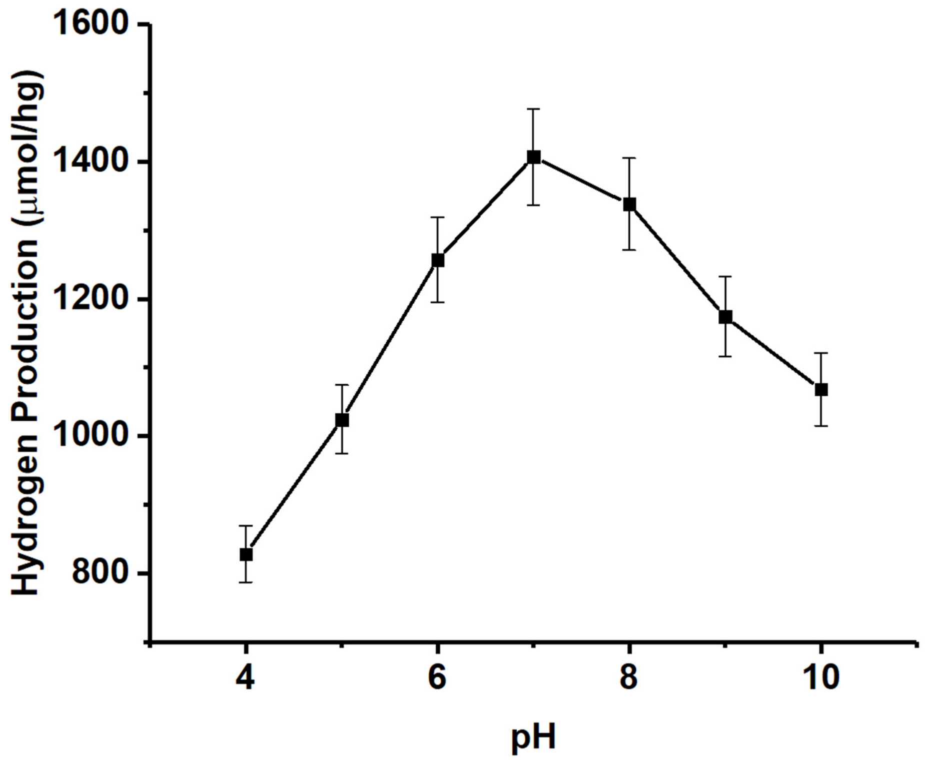

Figure S9. Effect of pH on the photocatalytic activity of the 5%(Co_3O_4-gC_3N_4)@ZnONPs catalyst for hydrogen production.

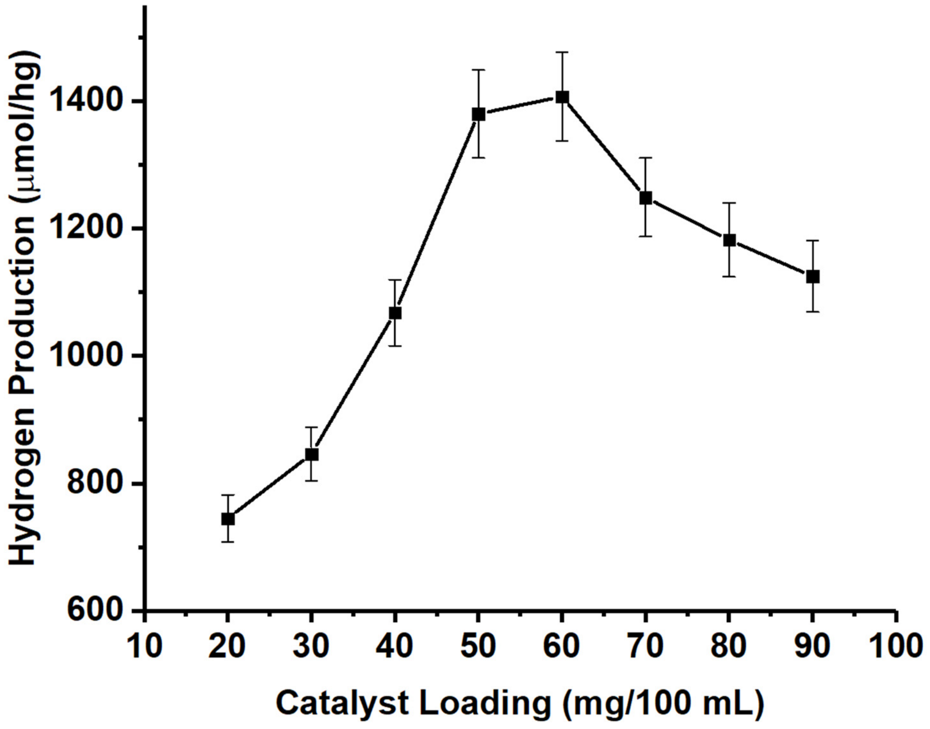

Figure S10. Evaluation of the initial concentration of 5%(Co_3O_4-gC_3N_4)@ZnONPs (**a**), on the efficiency of hydrogen production.

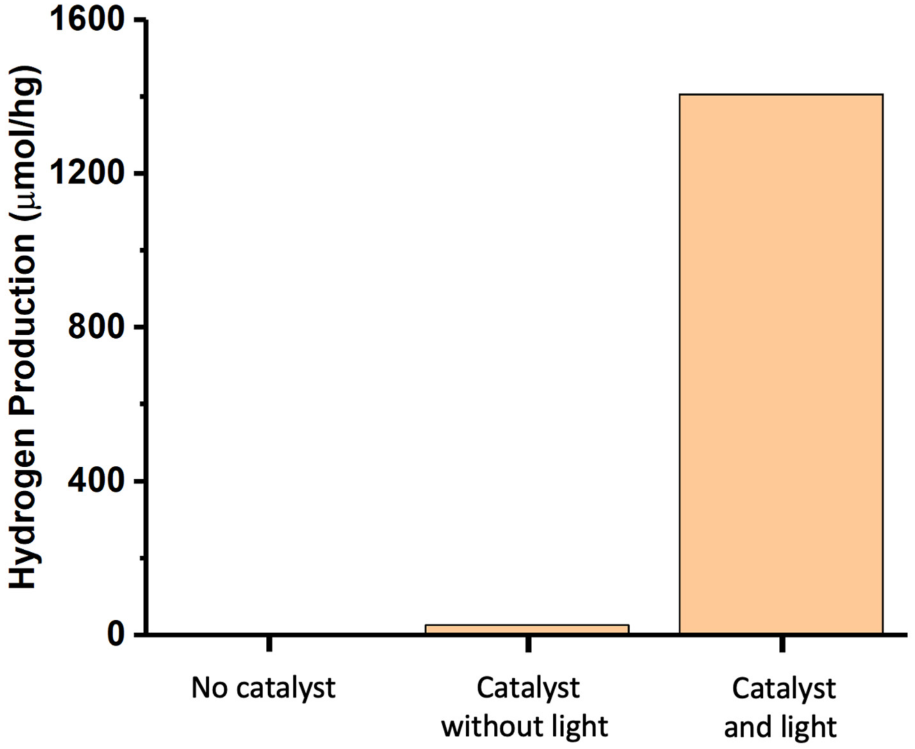

Figure S11. Control experiments for 5%(Co_3O_4-gC_3N_4)@ZnONPs on the efficiency of hydrogen production.

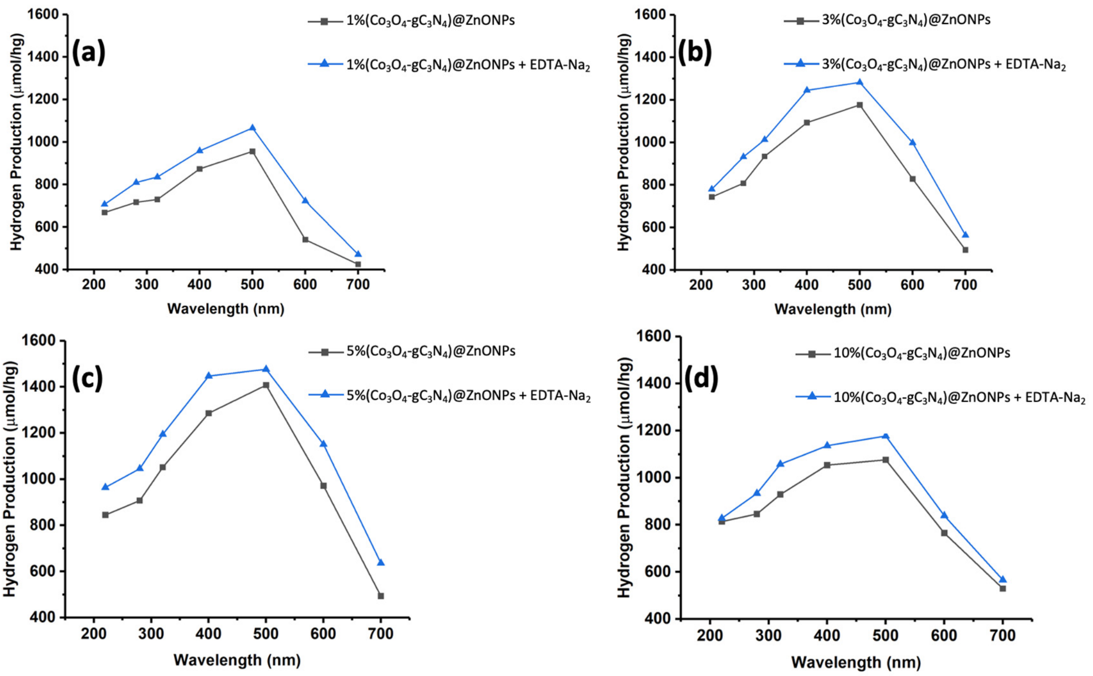

Figure S12. Hydrogen production via water splitting using various catalysts under irradiation, and also in the presence of a hole scavenger, namely EDTA-Na_2. 1%(Co_3O_4-gC_3N_4)@ZnONPs (**a**), 3%(Co_3O_4-gC_3N_4)@ZnONPs (**b**), 5%(Co_3O_4-gC_3N_4)@ZnONPs (**c**), and 10%(Co_3O_4-gC_3N_4)@ZnONPs (**d**).

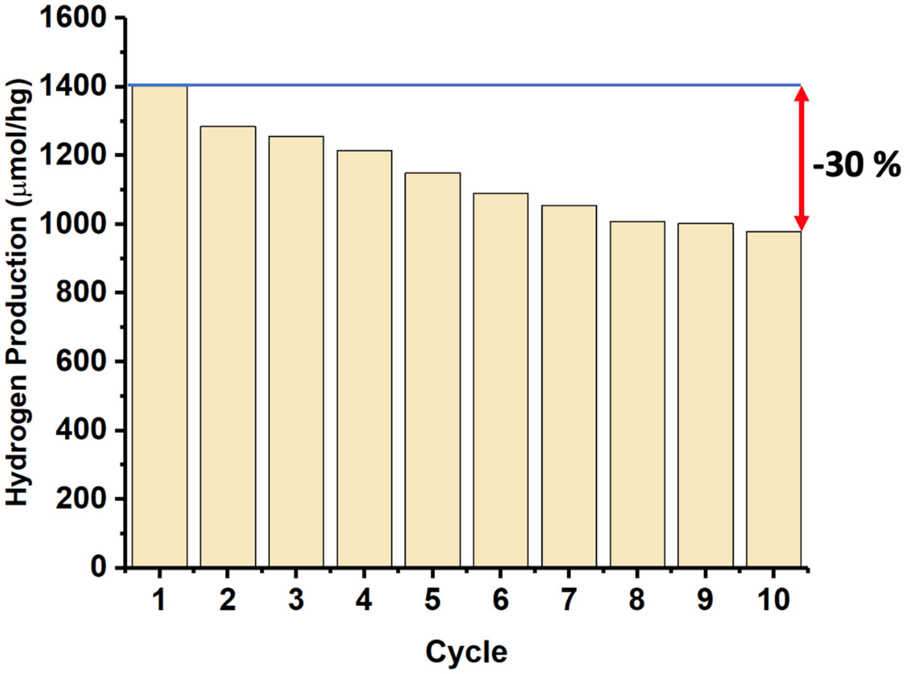

Figure S13. Recyclability of 5%(Co_3O_4-gC_3N_4)@ZnONPs after 10 consecutive catalytic cycles of hydrogen production, under irradiation at 500 nm.

Table S1. BET surface area of the as-synthesized materials.

Material	BET Area (m^2/g)
ZnONPs	76
Co_3O_4-gC_3N_4	187
1%(Co_3O_4-gC_3N_4)@ZnONPs	93
3%(Co_3O_4-gC_3N_4)@ZnONPs	127
5%(Co_3O_4-gC_3N_4)@ZnONPs	169
10%(Co_3O_4-gC_3N_4)@ZnONPs	231

Table S2. The pseudo-first-order kinetics constants for the photodegradation of CFX using the as-synthesized catalysts.

Catalyst	Apparent Rate[1,2]	Error
ZnONPs	0.0189	0.0002
1%gC_3N_4@ZnONPs	0.0223	0.0004
3%gC_3N_4@ZnONPs	0.0267	0.0002
5%gC_3N_4@ZnONPs	0.0292	0.0003
10%gC_3N_4@ZnONPs	0.0341	0.0005
1%(Co_3O_4-gC_3N_4)@ZnONPs	0.0306	0.0005
3%(Co_3O_4-gC_3N_4)@ZnONPs	0.0388	0.0005
5%(Co_3O_4-gC_3N_4)@ZnONPs	0.0404	0.0003
10%(Co_3O_4-gC_3N_4)@ZnONPs	0.0702	0.0019

[1] (k, min^{-1}). [2] (R^2 was 0.99 in all cases)

Table S3. Maximum H_2 production (μmol/hg) by the different catalysts and precursors, under irradiation at several wavelengths. All values obtained are affected by 5% error.

Catalyst	Irradiation Wavelength (nm)						
	220	280	320	400	500	600	700
ZnONPs	476	428	380	349	298	179	116
Co_3O_4-gC_3N_4	450	428	422	525	537	379	171
gC_3N_4@ZnONPs	436	387	312	370	393	227	191
1%(Co_3O_4-gC_3N_4)@ZnONPs	669	716	729	873	956	540	425
3%(Co_3O_4-gC_3N_4)@ZnONPs	742	808	931	1093	1176	828	493
5%(Co_3O_4-gC_3N_4)@ZnONPs	845	907	1052	1286	1407	972	492
10%(Co_3O_4-gC_3N_4)@ZnONPs	814	846	929	1053	1076	765	529
